# Supplementary figures and images for: A daily diary study on adolescents’ mood, empathy, and prosocial behavior during the COVID-19 pandemic
Source: PLoS One. 2020 Oct 7;15(10):e0240349. doi: 10.1371/journal.pone.0240349 (PMC7540854; doi:10.1371/journal.pone.0240349)

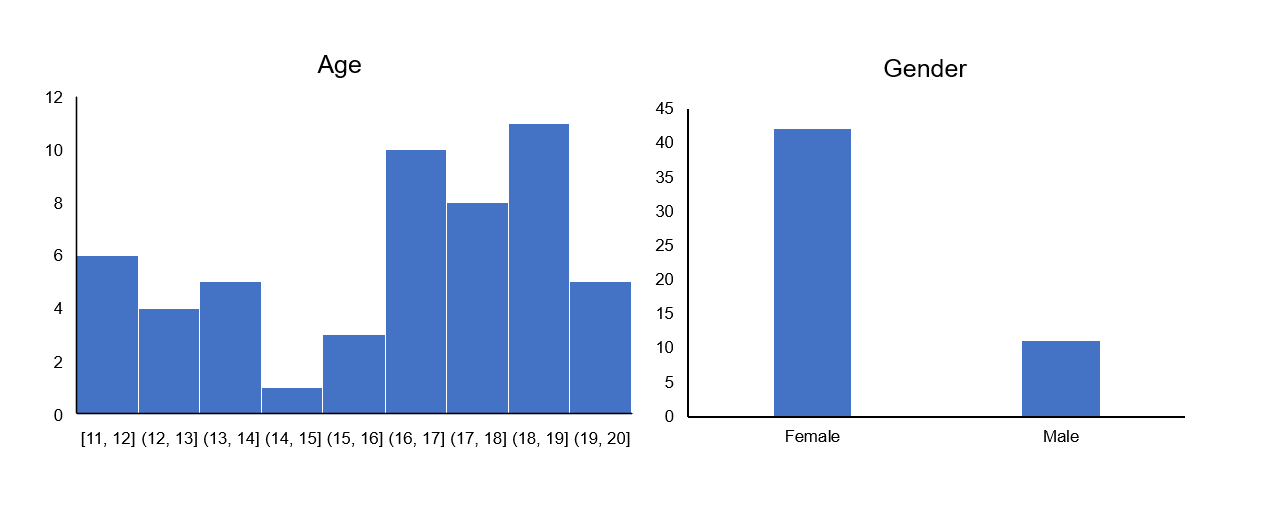

Supplement: S1 Fig — (TIF) [file pone.0240349.s001.tif]
