# Supplementary material for: A daily diary study on adolescents’ mood, empathy, and prosocial behavior during the COVID-19 pandemic
Source: PLoS One. 2020 Oct 7;15(10):e0240349. doi: 10.1371/journal.pone.0240349 (PMC7540854; doi:10.1371/journal.pone.0240349)
Supplement: S1 File — (DOCX) [file pone.0240349.s002.docx]

**S1. Details regarding statistical analyses, including data checks.**

**Assumption checks.** To address our research questions, we performed correlations and Generalized Estimating Equations. Beforehand, we checked data with regard to normality outliers, and linearity. The perspective taking-, empathic concern-, GCS-, emotional support-, vigor- and tension-scores showed no deviations from normality as indicated by Shapiro-Wilk tests (*n.s.*), and no outliers (i.e., standardized residuals < -3.29, > 3.29); except from slight deviations from normality for perspective taking at T1 (Shapiro-Wilk, *p* = .040), emotional support at T2 (Shapiro-Wilk, *p* = .030) and dire prosociality at T1 (Shapiro-Wilk, *p* = .009). For giving, altruistic prosociality, and Social Value Orientation (SVO), none of the scores were normally distributed (Shapiro-Wilk tests, *p*’s < .020), except from altruistic prosociality at week 2 (Shapiro-Wilk test *p* = .056). However, as transformations hamper interpretability and barely improved the normality of the data and most of the statistical tests ran here are relatively robust against violations of normality, we decided to nevertheless report tests using a normal distribution. With regard to giving scores, three individuals were outliers: one on giving to a friend on day 1 (10 coins, z-score = 5.48), one on giving to an unfamiliar peer on day 15 (0 coins, z-score -3.34), and one on giving to a friend on day 15 (10 coins, z-score 4.41). With regard to dire prosociality, there were no outliers, and with regard to altruistic prosociality there two individuals were outliers: one at T1 (score 1.83, z-score -4.15) and one at T2 (score 2.00, z-score -4.30). With regard to SVO, there were two outliers, one at T2 (7.82, z-score -3.50) and one on day 1 of the pandemic daily diary study (7.82, z-score -6.67). However, because these outliers reflect behavior of interest and there was no reason to assume that participants misunderstood the task or instructions, we decided to retain these outliers in the dataset. Scatterplots of the associations between perspective taking, empathic concern, and GCS-scores on the one hand and giving scores on the others hand showed no evidence that associations were non-linear.
