# Supplementary material for: A daily diary study on adolescents’ mood, empathy, and prosocial behavior during the COVID-19 pandemic
Source: PLoS One. 2020 Oct 7;15(10):e0240349. doi: 10.1371/journal.pone.0240349 (PMC7540854; doi:10.1371/journal.pone.0240349)
Supplement: S2 File — (DOCX) [file pone.0240349.s003.docx]

**S2. Results for other, pandemic-only measures assessed during the daily diary study.**

As described in detail on the Open Science Framework (<https://osf.io/5ejhc/>) we assessed several other measures only during the COVID-19 pandemic daily diary study, including risk propensity, and several questions specifically related to the pandemic. Apart from one exception, no changes in these measures were found over the course of the pandemic. The exception concerned ‘I ruminate a lot about what’s happening in the world’, for which a repeated measures ANOVA (*N* = 38) with time (week 1, 2, and 3) as within-subject factor showed a main effect of time, *F*(2, 74) = 4.48, *p* = .018, *η*²_p_ = .11. Bonferroni corrected pairwise comparisons showed that participants reported higher levels of rumination in week 1 (*M* = 2.85, *SD* = 1.48) compared to week 2 (*M* = 2.45, *SD* = 1.47). No difference was found between week 1 and 3 and 2 and 3 (*M* = 2.50, *SD* = 1.54).
