# Supplementary material for: A daily diary study on adolescents’ mood, empathy, and prosocial behavior during the COVID-19 pandemic
Source: PLoS One. 2020 Oct 7;15(10):e0240349. doi: 10.1371/journal.pone.0240349 (PMC7540854; doi:10.1371/journal.pone.0240349)
Supplement: S3 File — (DOCX) [file pone.0240349.s004.docx]

**S3. Histograms of key variables**


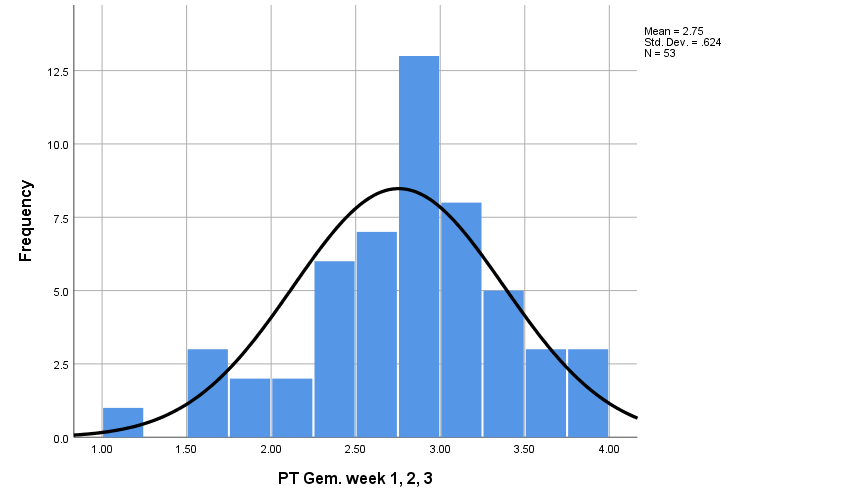


Perspective Taking
 (average week 1, 2, 3 pandemic)


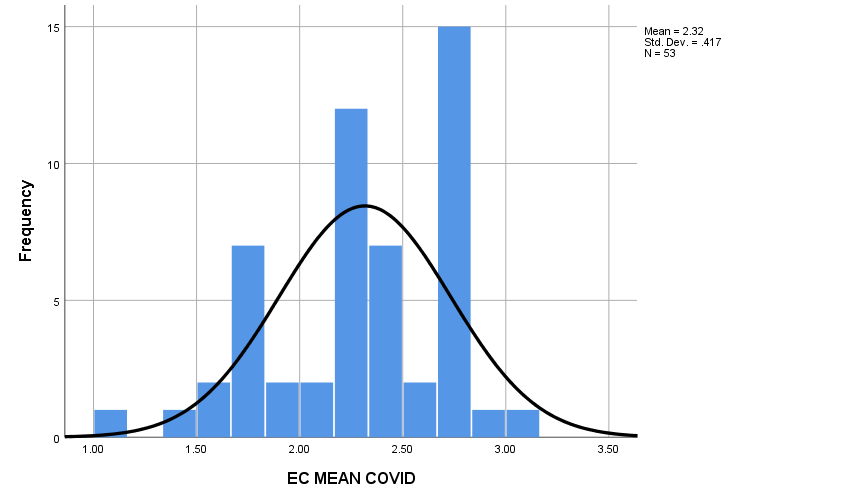


Empathic Concern
 (average week 1, 2, 3 pandemic)


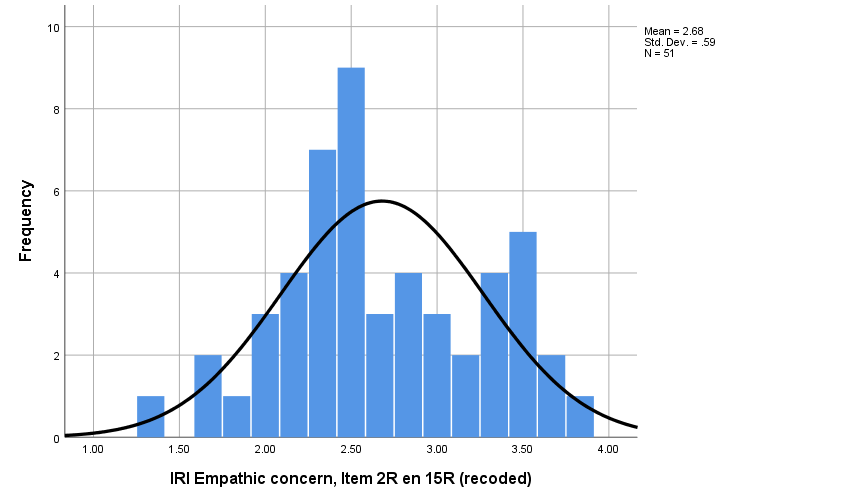


Empathic Concern
 T1


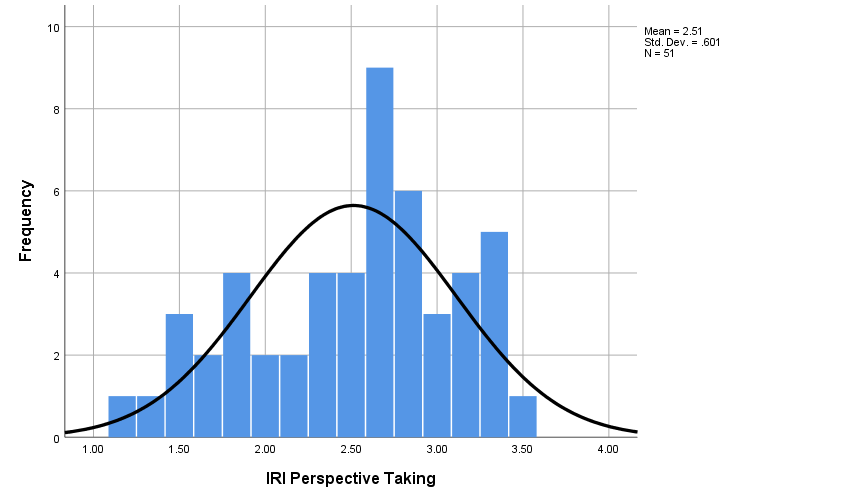


Perspective Taking
 T1


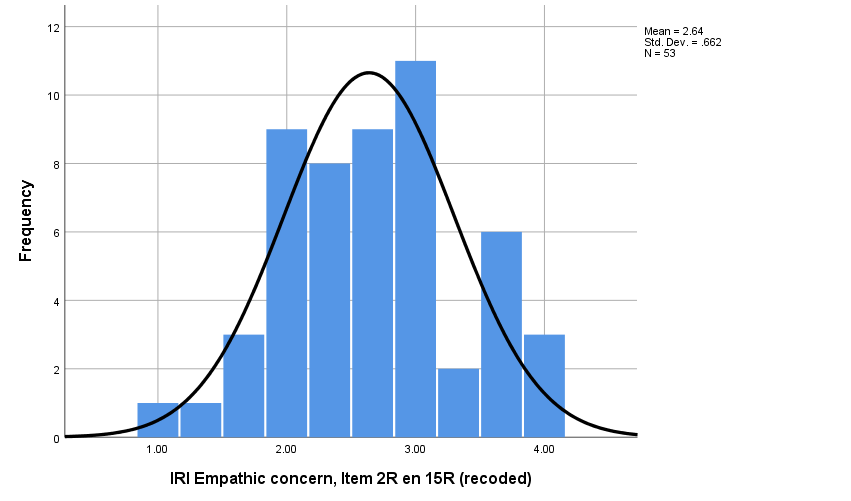


Empathic Concern
 T2


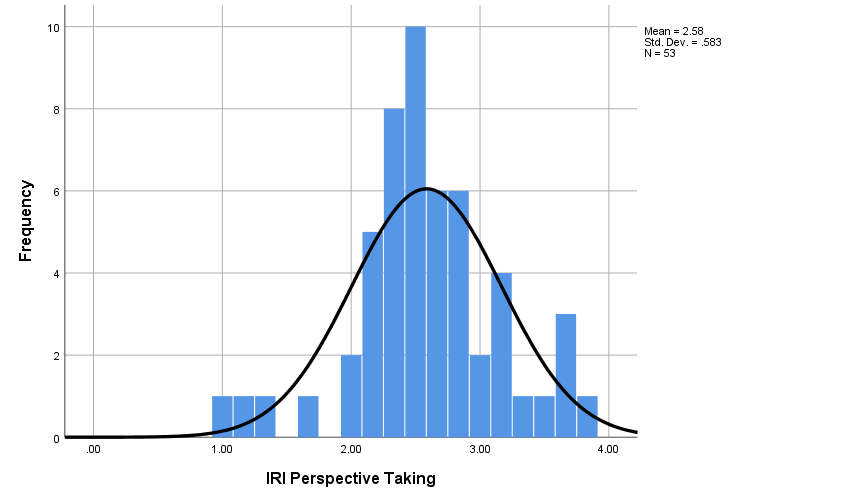


Perspective Taking
 T2


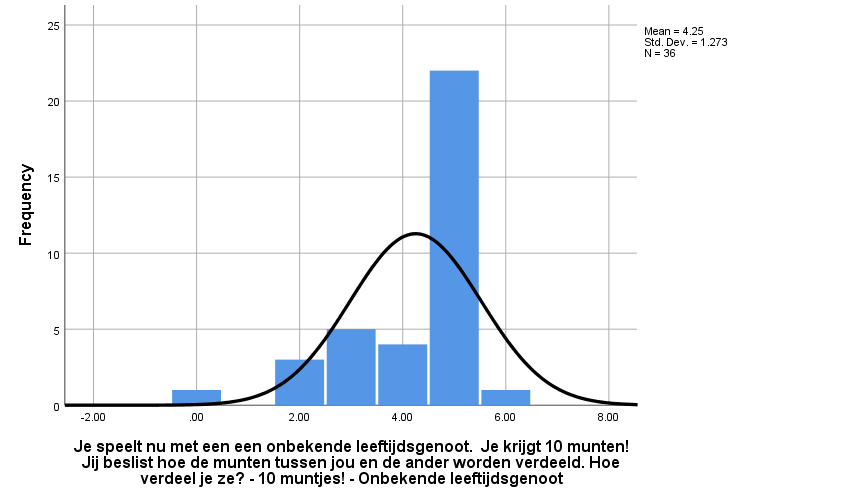


Giving to Unfamiliar Peer
Day 15


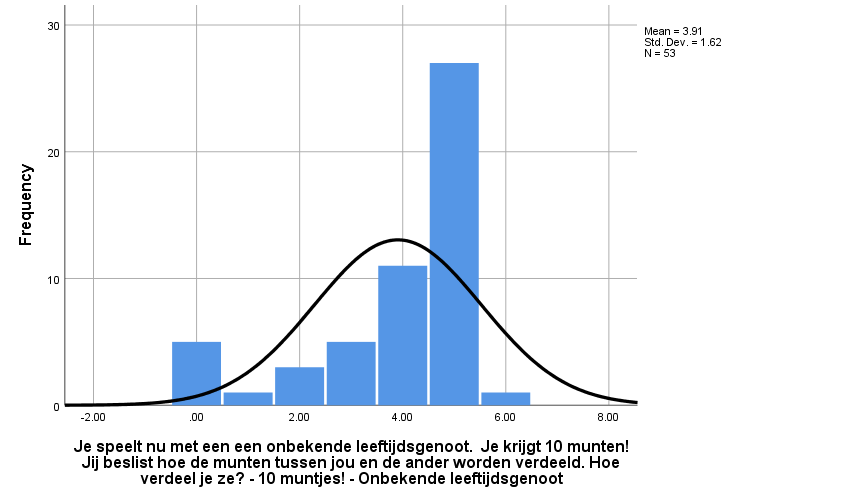


Giving to Unfamiliar Peer
Day 1


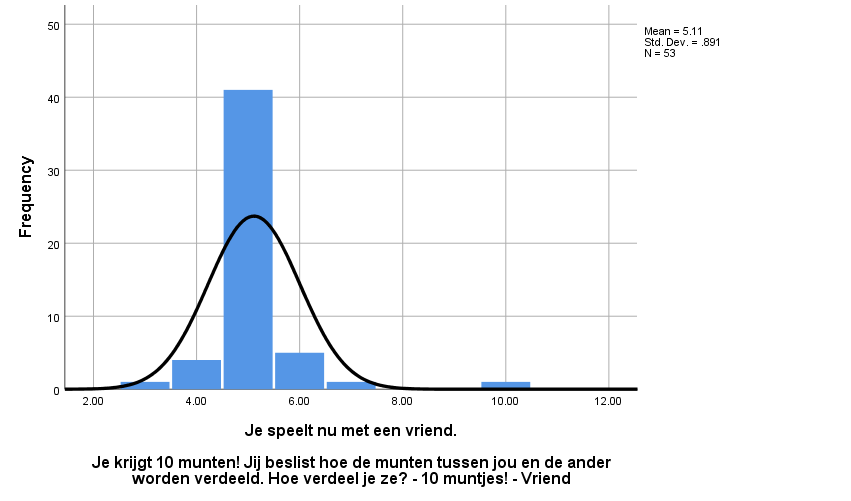


Giving to Friend
Day 1


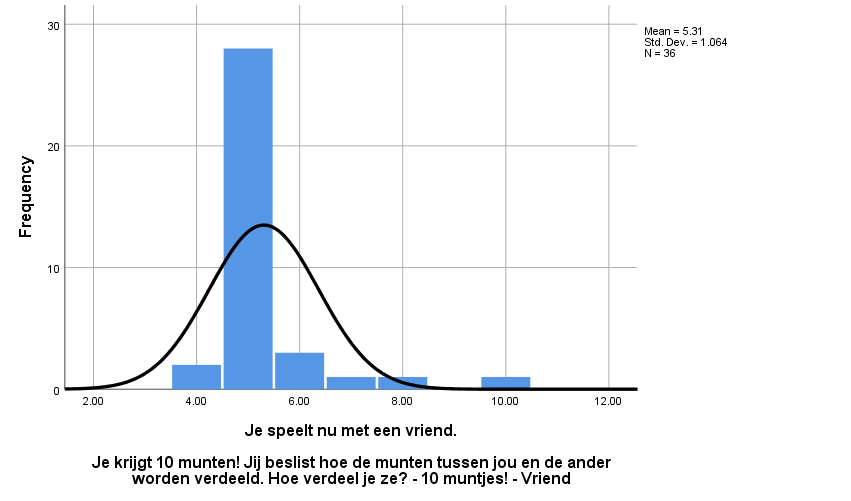


Giving to Friend
Day 15


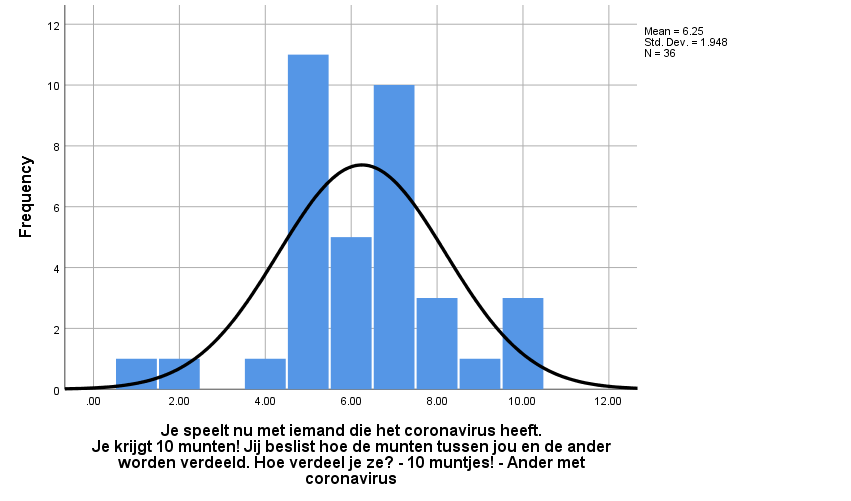


Giving to Individual with COVID-19
Day 15


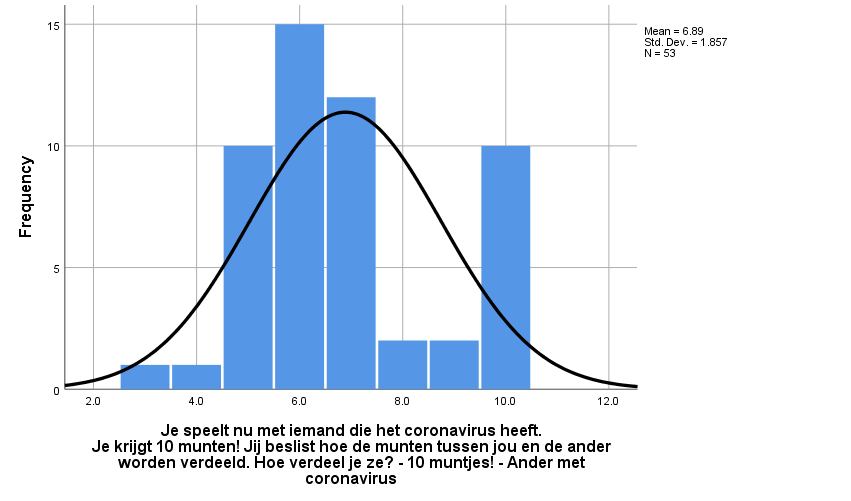


Giving to Individual with COVID-19
Day 1


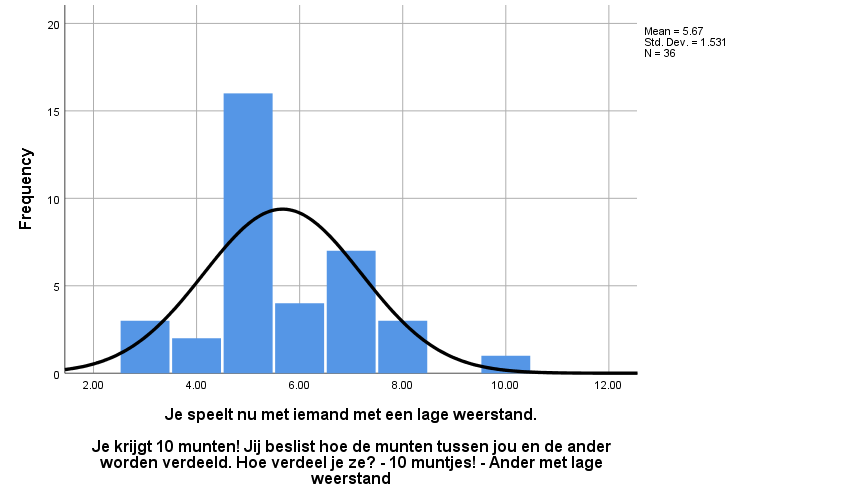


Giving to Individual with Poor Immune System
Day 15


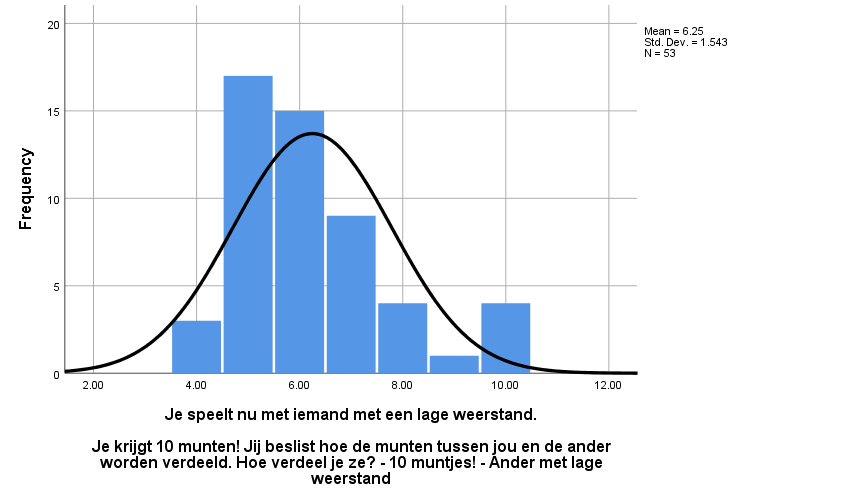


Giving to Individual with Poor Immune System
Day 1


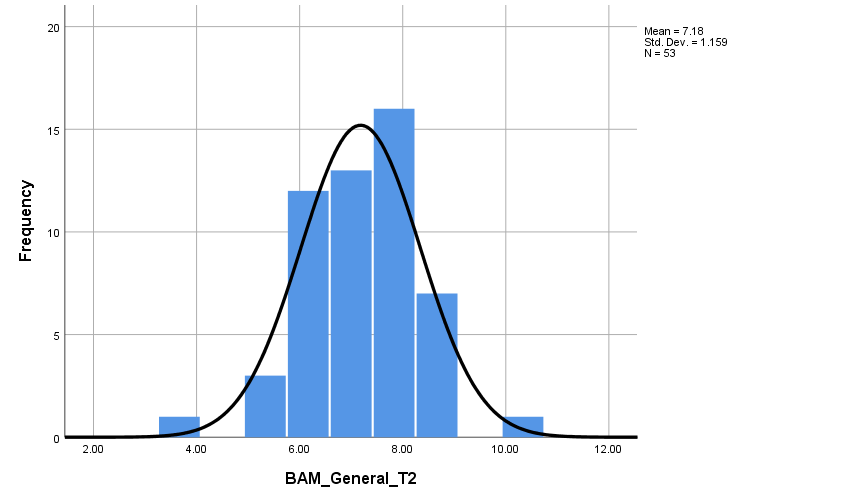


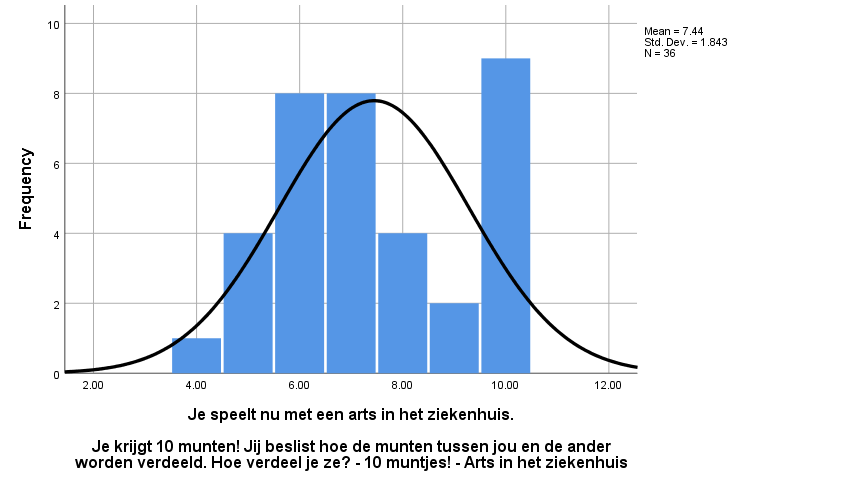


Giving to Doctor in Hospital
Day 15


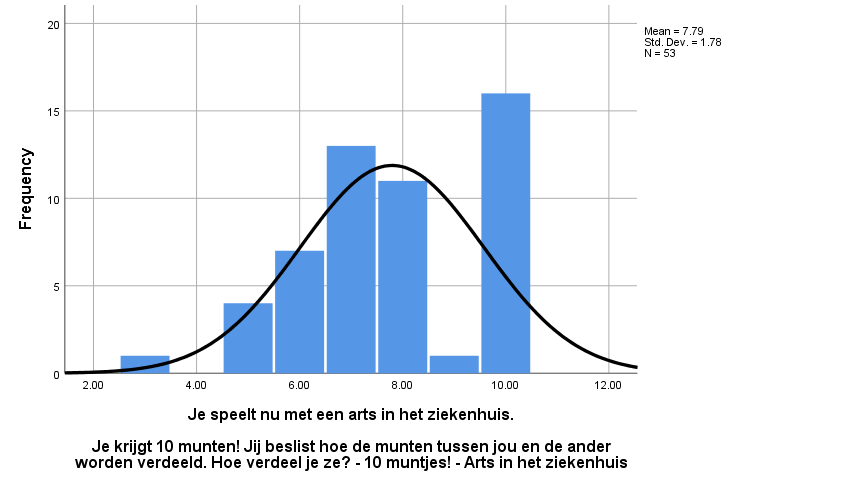


Giving to Doctor in Hospital
Day 1


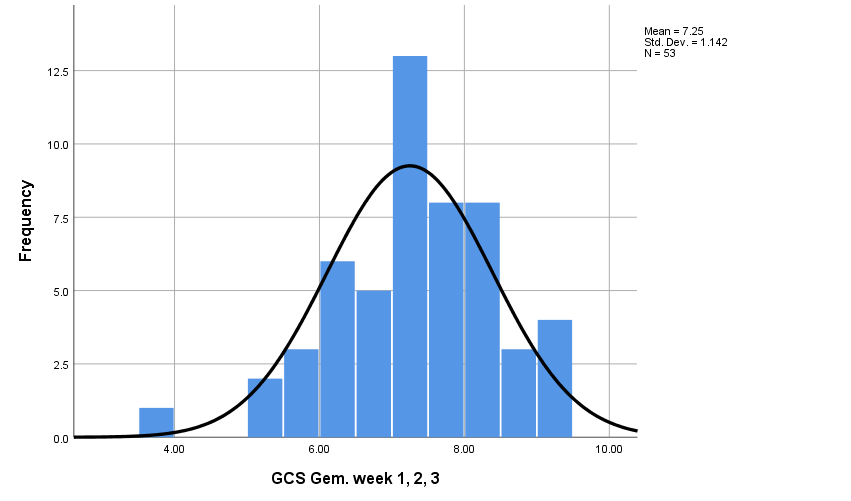


General contributions to society
(average pandemic week 1, 2, 3)


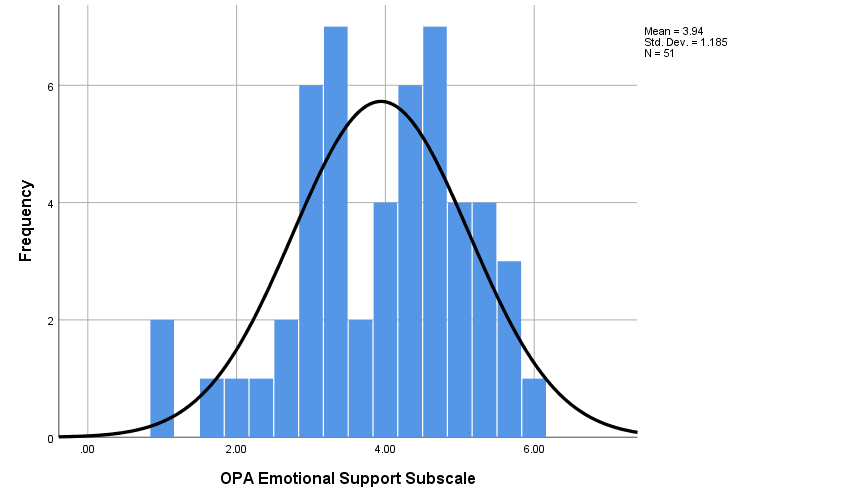


Opportunities for prosocial actions: emotional support
T1

General contributions to society
(average pandemic T2)


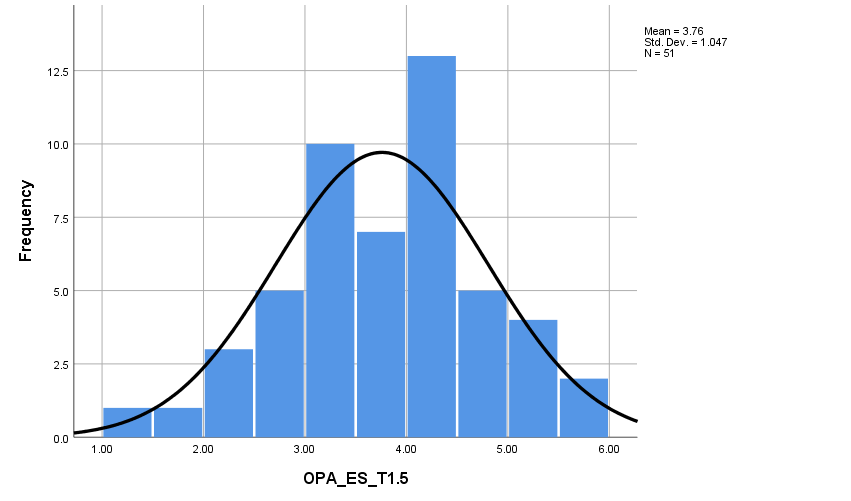


Opportunities for prosocial actions: emotional support
T1.5


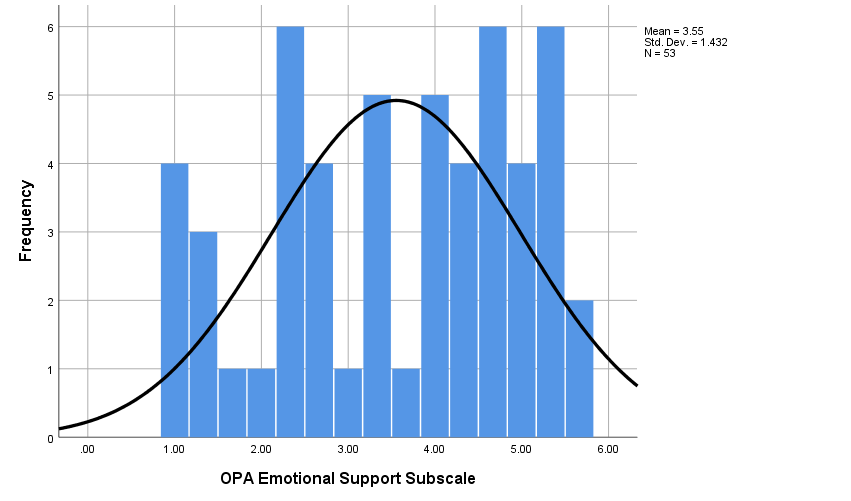


Opportunities for prosocial actions: emotional support
T2


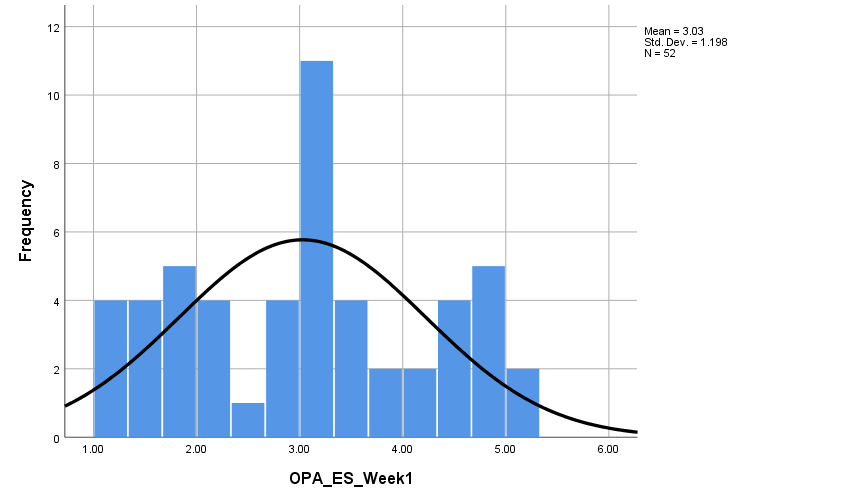


Opportunities for prosocial actions: emotional support
Week 1 pandemic


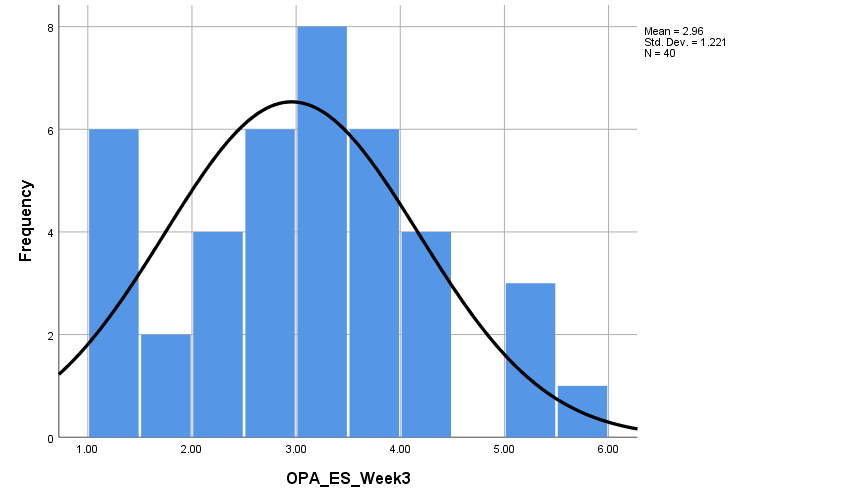


Opportunities for prosocial actions: emotional support
Week 3 pandemic


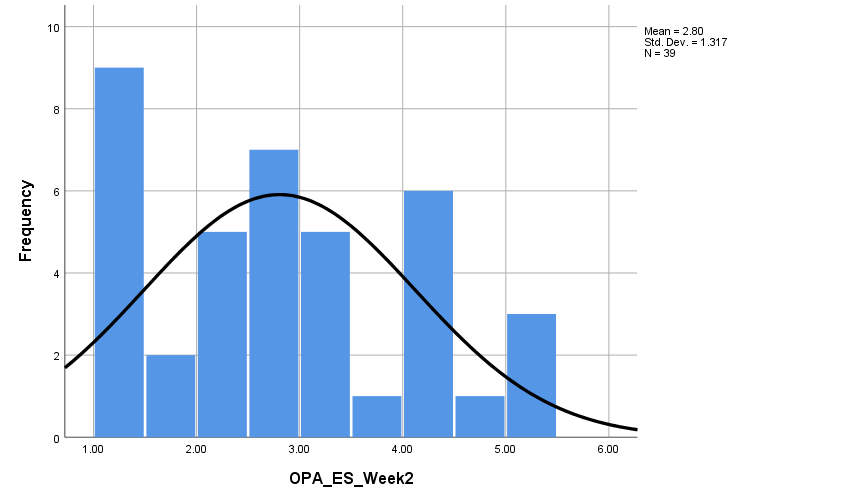


Opportunities for prosocial actions: emotional support
Week 2 pandemic


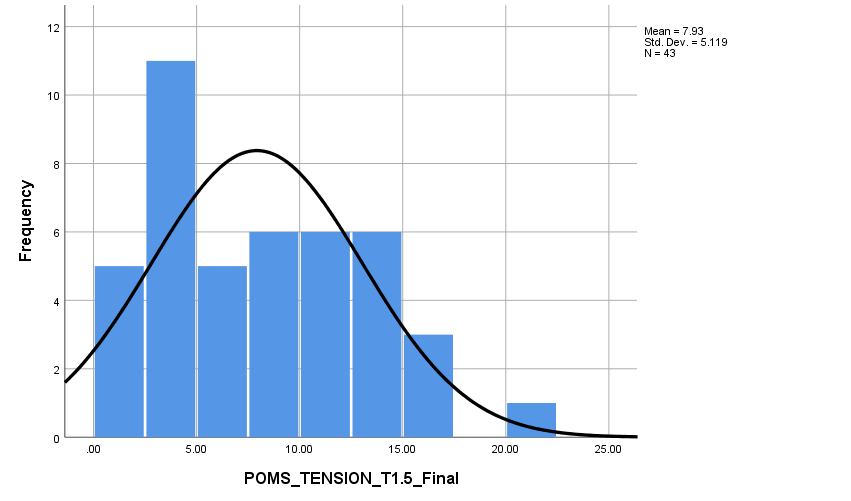


Tension
T1.5


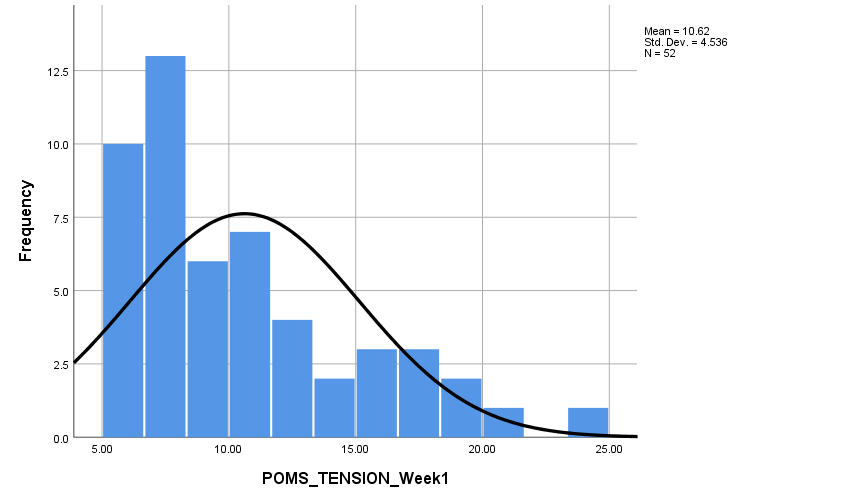


Tension
Week 1 pandemic


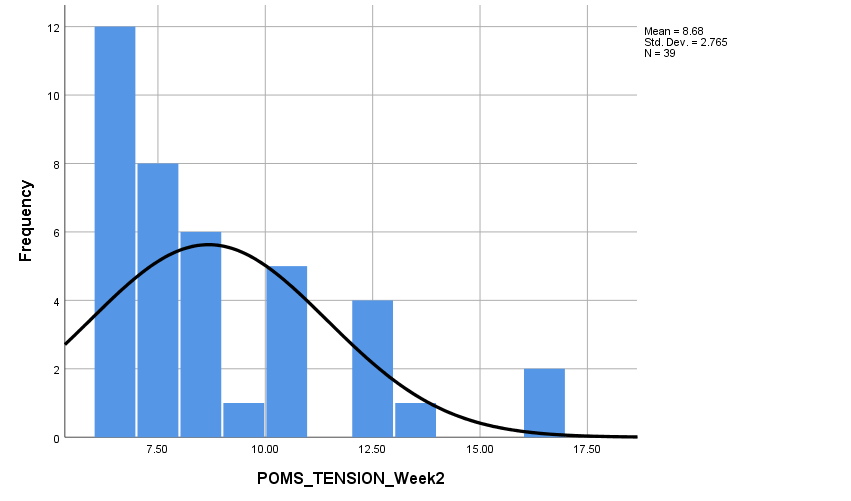


Tension
Week 2 pandemic


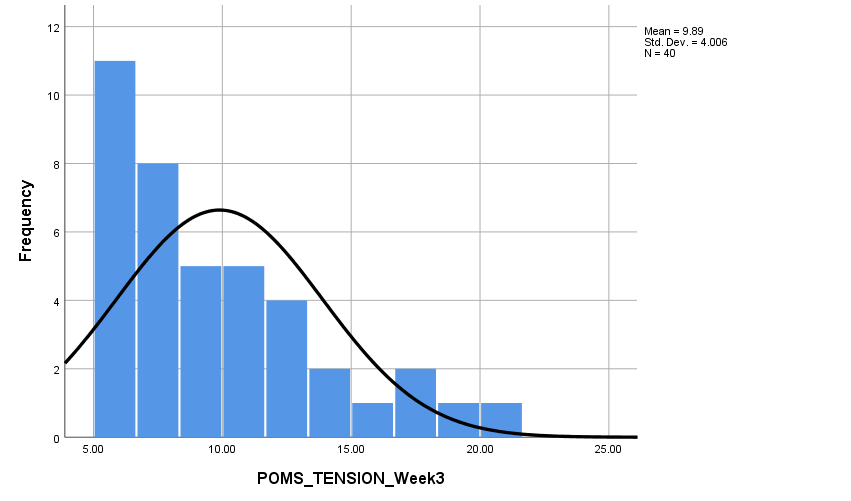


Tension
Week 3 pandemic


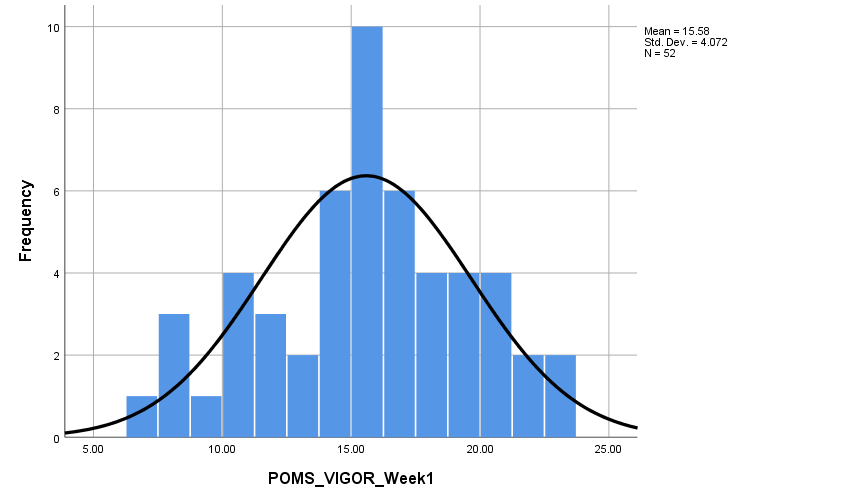


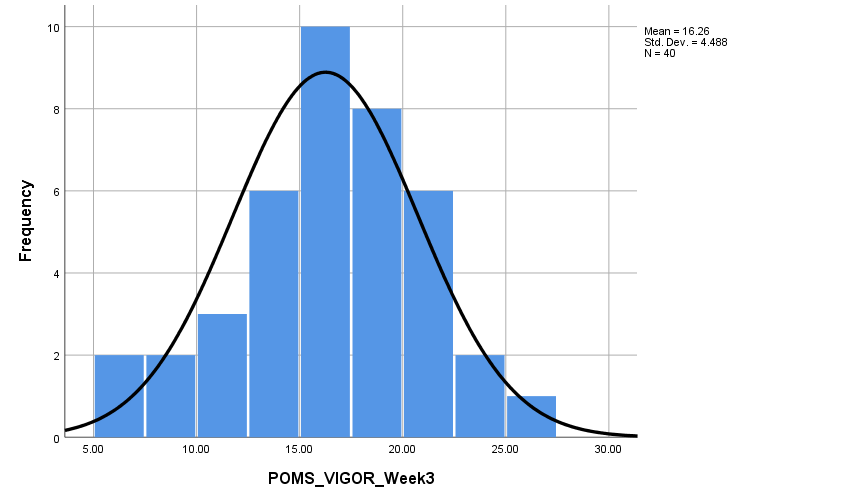


Vigor
Week 3 pandemic


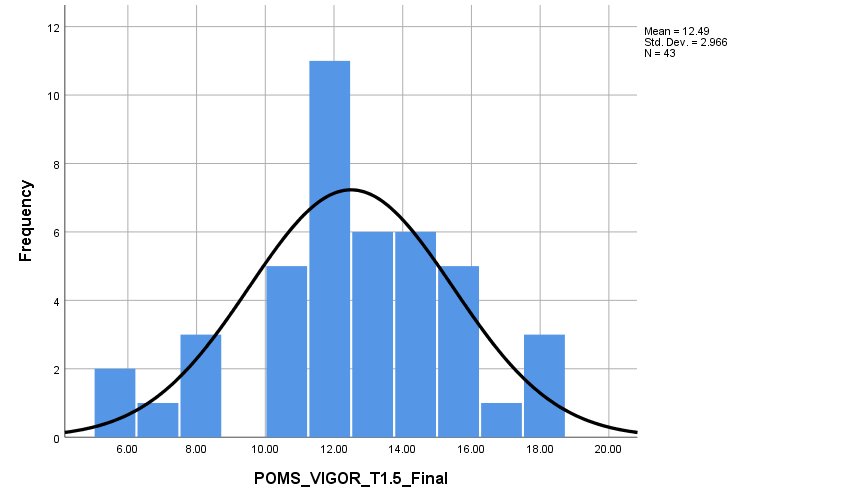


Vigor
T1.5


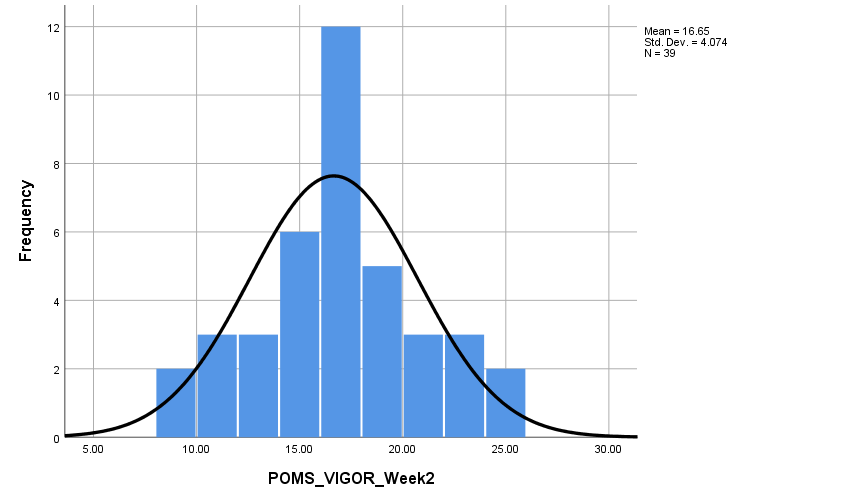


Vigor
Week 2 pandemic


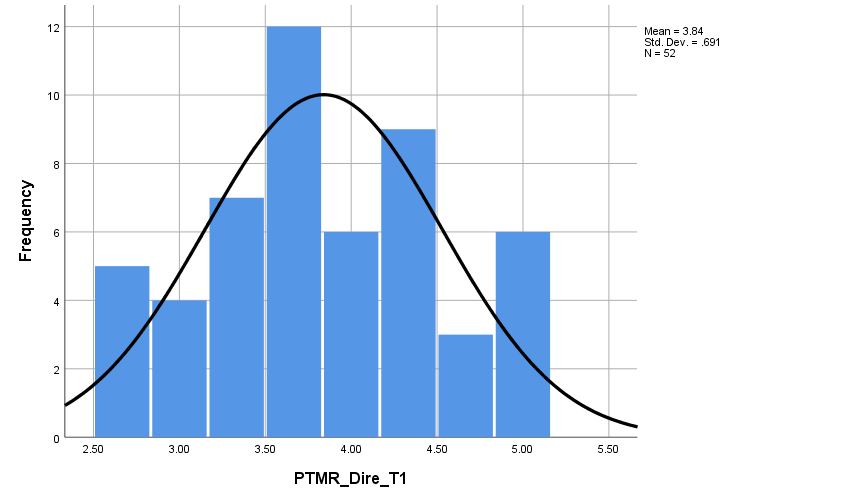


Prosocial Tendencies Measure Revised: Dire Prosociality
T1


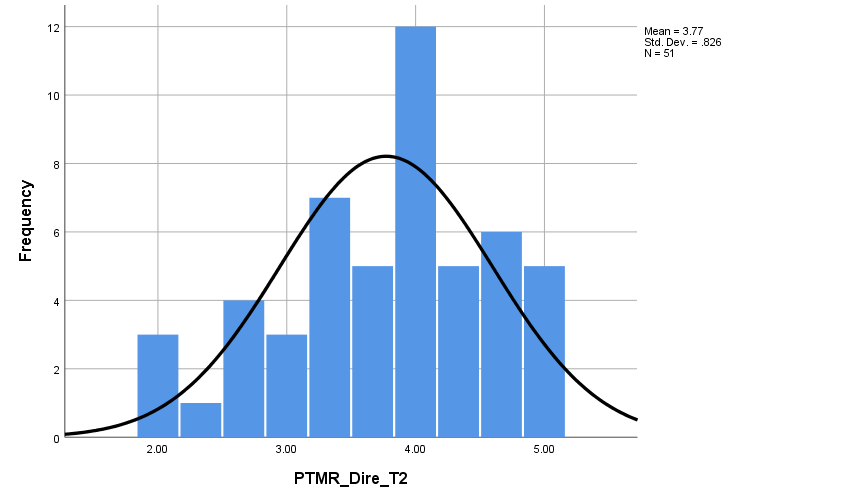


Prosocial Tendencies Measure Revised: Dire Prosociality
T2

Vigor
Week 1 pandemic


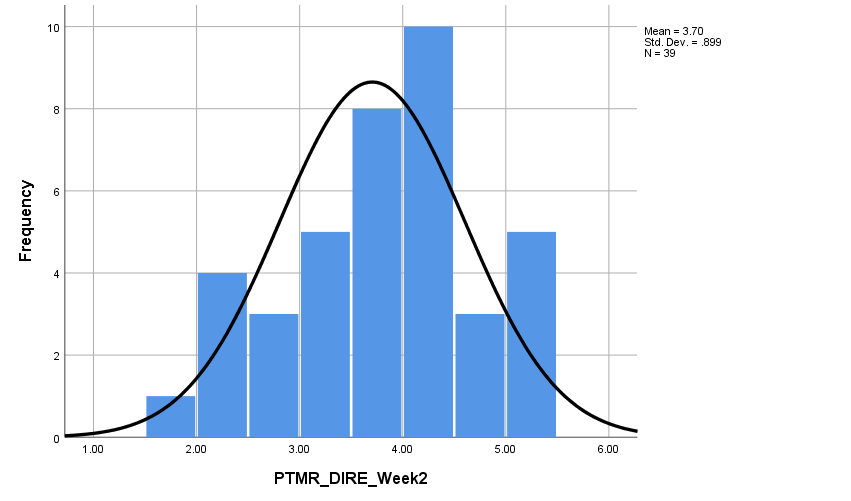


Prosocial Tendencies Measure Revised: Dire Prosociality
Week 2 pandemic


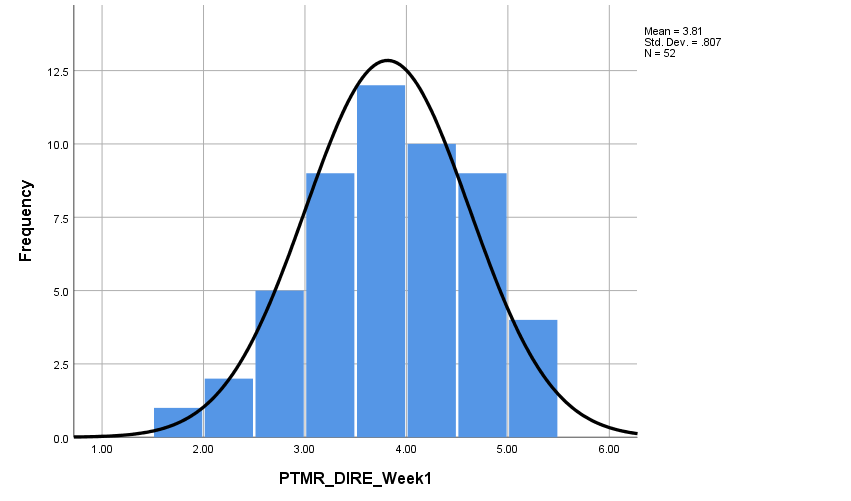


Prosocial Tendencies Measure Revised: Dire Prosociality
Week 1 pandemic


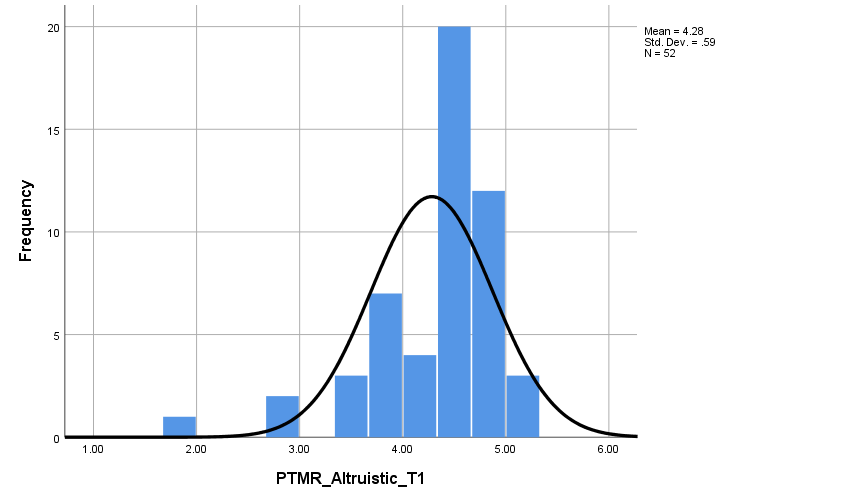


Prosocial Tendencies Measure Revised: Altruistic Prosociality
T1


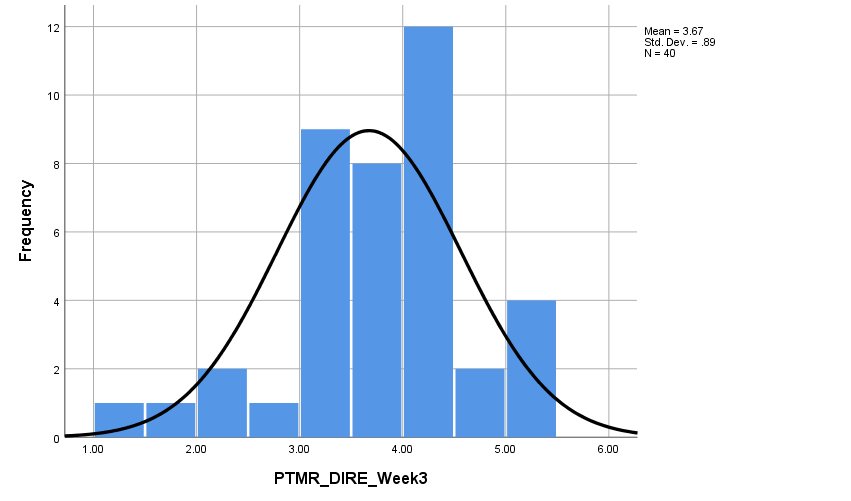


Prosocial Tendencies Measure Revised: Dire Prosociality
Week 3 pandemic


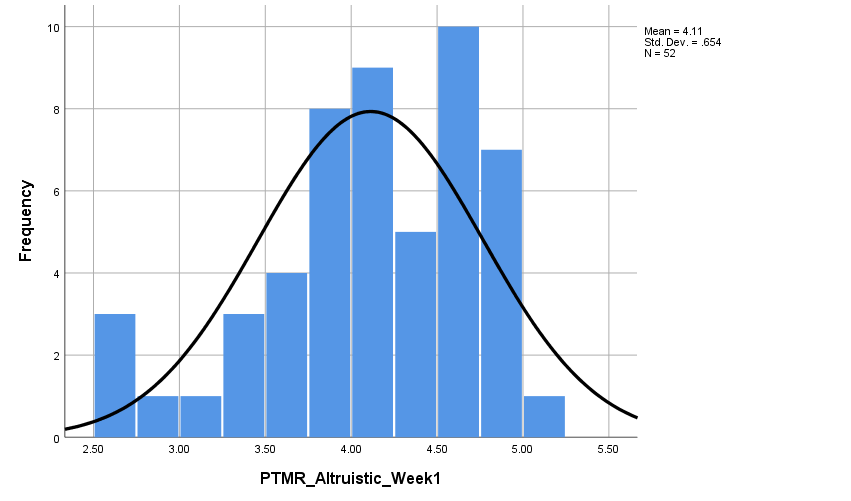


Prosocial Tendencies Measure Revised: Altruistic Prosociality
Week 1 pandemic


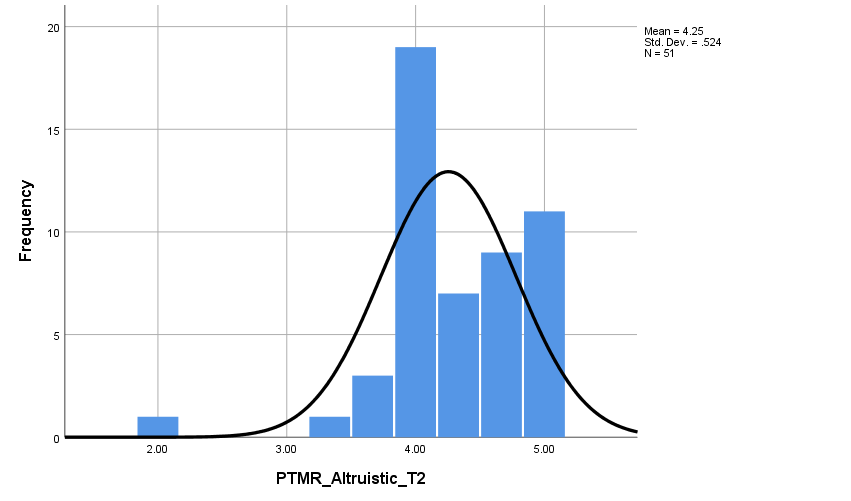


Prosocial Tendencies Measure Revised: Altruistic Prosociality
T2


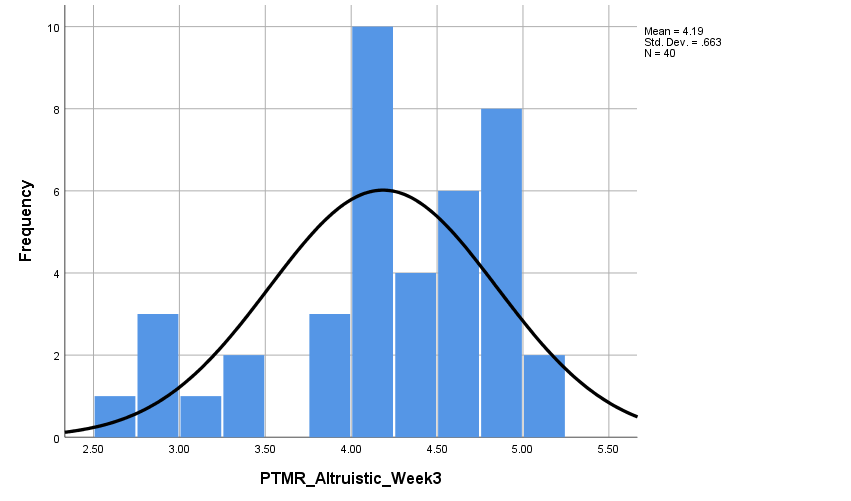


Prosocial Tendencies Measure Revised: Altruistic Prosociality
Week 3 pandemic


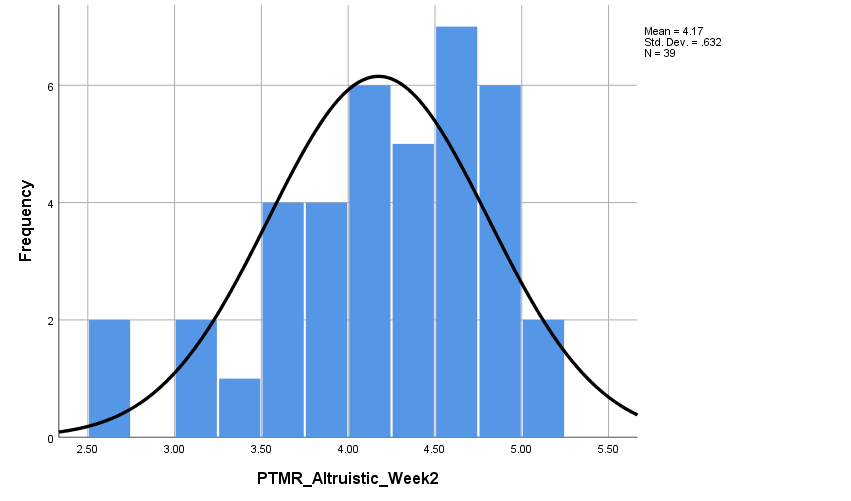


Prosocial Tendencies Measure Revised: Altruistic Prosociality
Week 2 pandemic


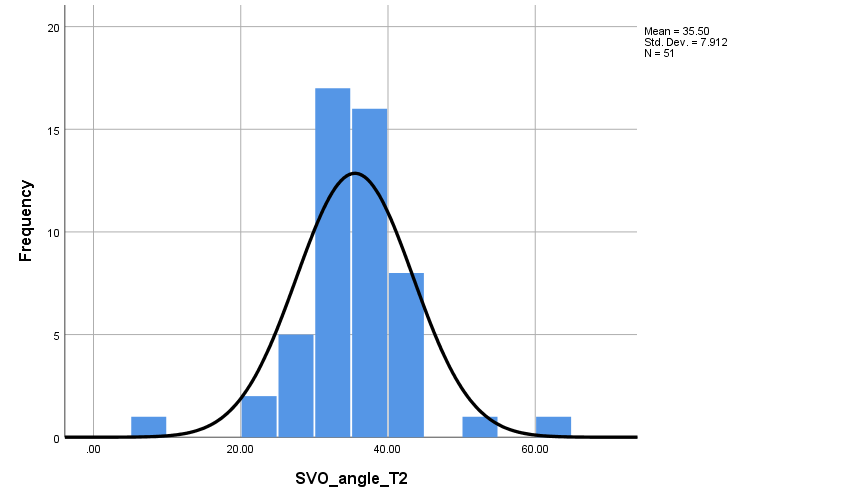


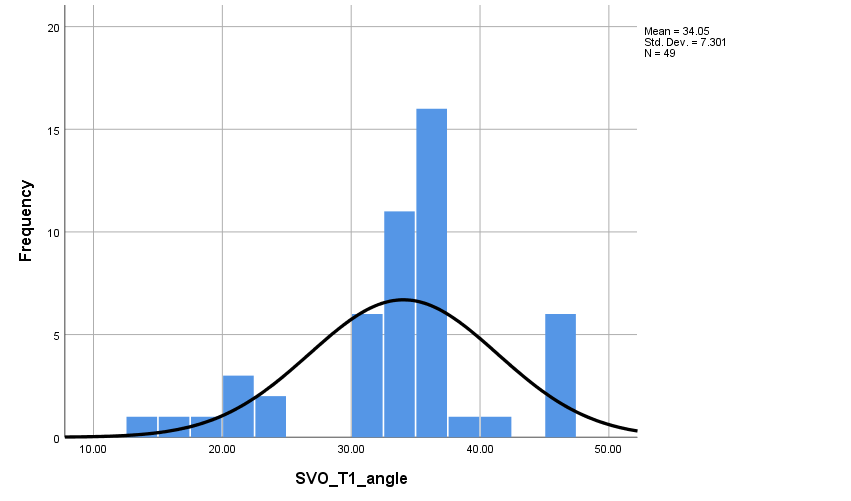


Social Value Orientation Angle
T1


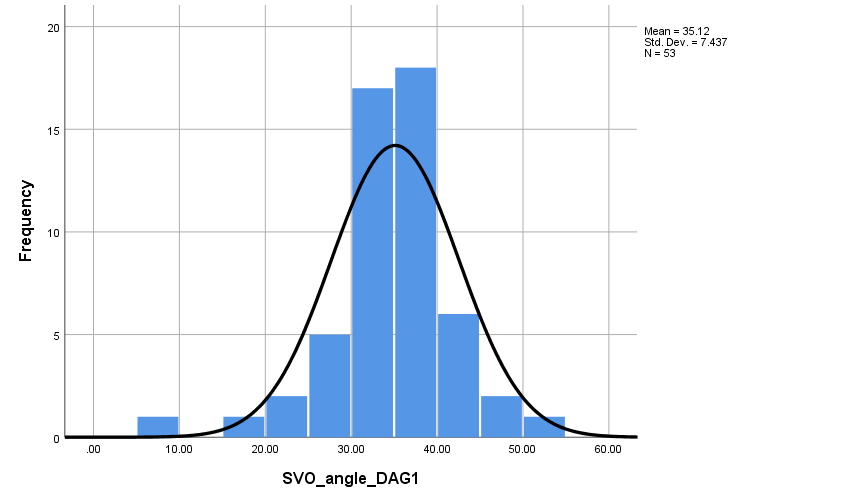


Social Value Orientation Angle
Day 1 Daily Diary Study Pandemic


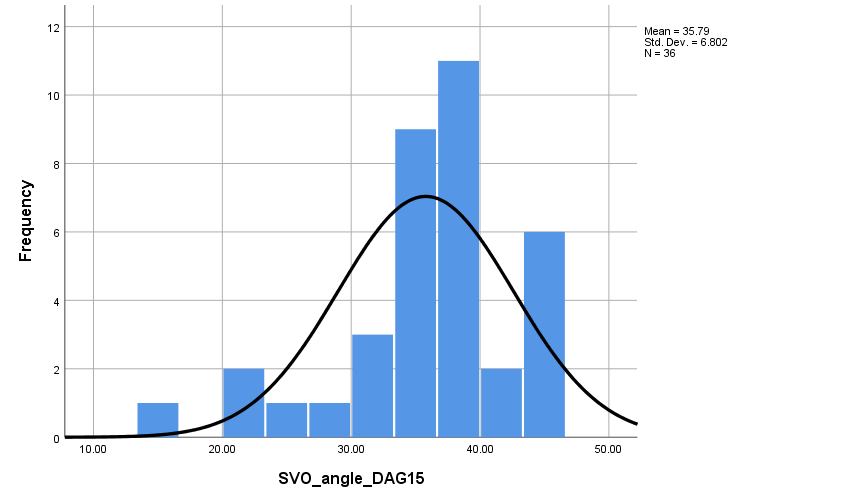


Social Value Orientation Angle
Day 15 Daily Diary Study Pandemic
