# Supplementary material for: A daily diary study on adolescents’ mood, empathy, and prosocial behavior during the COVID-19 pandemic
Source: PLoS One. 2020 Oct 7;15(10):e0240349. doi: 10.1371/journal.pone.0240349 (PMC7540854; doi:10.1371/journal.pone.0240349)
Supplement: S4 File — (DOCX) [file pone.0240349.s005.docx]

**Overview of measures for the project ‘Prosocial Behavior in Adolescence During the Pandemic Covid-19 Crisis’**

*The current document contains all questionnaires and measures that we administered for our project ‘Prosocial Behavior in Adolescence During the Pandemic Covid-19 Crisis’. Please note that questionnaires were administered to our participants in Dutch but were translated to English here for sharing purposes. The Dutch version of the questionnaires can found in another document on our OSF page. If you would like to use any of the measures described here, please give credit to the original authors. For the measures that we developed ourselves (e.g. pandemic questionnaire, questionnaires about contributing to society), we ask you to give us credit by referring to our OSF page:* [*https://osf.io/kgcdm/*](https://osf.io/kgcdm/)*.*

# **Daily Measures**

1. **Opportunities for Prosocial Actions – Emotional Support Subscale***Questionnaire not yet validated, adjusted to match pandemic situation*

We would like to know more about the things you did for your friends last day. This does not include someone with whom you have a romantic relationship.

You will get questions about multiple things you could do for someone else. Here we do not refer to activities you would get payed for, or you would be punished for if you would not perform them. We are only interested in what you did by yourself.

Please fill out below whether you performed the activities for your friends last day.

1. I comforted a friend last day
2. Last day, I did my best to spend time with friends
3. Last day, I send a message to a friend

*Fillled out using a Likert Scale: 1) Not at all – 5) A lot*

1. **Prosocial Tendencies Measure Revised – Altruism Subscale***Questionnaire developed by Carlo et al. (2003), adjusted by us to match pandemic situation*

Below you can find sentences that can either describe you or not. Please indicate how much every sentence applies to you by using the following scale. 1) Does not describe me at all, 2) describes me a little, 3) describes me reasonably, 4) describes me well, 5) describes me very well.

1. I think that one of the best things about helping others is that it makes me look good. **R**
2. I believe that helping others works best when I get some benefit. **R**
3. One of the best things about helping others is that it makes me look good. **R**
4. I feel that if I help someone, they should help me in the future. **R**
5. I believe I should receive more rewards for the time and energy I spend helping others. **R**
6. I often help even if I don’t think I will get anything out of helping.
7. **Prosocial Tendencies Measure Revised – Dire Subscale***Questionnaire developed by Carlo et al. (2003)*

Below you can find sentences that can either describe you or not. Please indicate how much every sentence applies to you by using the following scale. 1) Does not describe me at all, 2) describes me a little, 3) describes me reasonably, 4) describes me well, 5) describes me very well.

1. I tend to help people who are in a real crisis or need.
2. I tend to help people who are hurt badly.
3. It is easy for me to help others when they are in a bad situation.
4. **Pandemic Questionnaire***Self-developed by van de Groep, Sweijen, Green, Zanolie & Crone (2020)*
5. Are you currently in self-isolation? That means that you do not leave your house and do not have any physical contact with the outside world.
   *Possible answers: 1) Yes, 2) No, 3) Other: …*
6. Indicate whether you agree with the following statement. I feel that at this moment, in this crisis, I can be of use to society.
   *Possible answers: 1) Entirely agree, 2) Mostly Agree, 3) Somewhat agree, 4) Neither Agree nor Disagree, 5) Somewhat Disagree, 6) Mostly Disagree, 7) Entirely disagree*
7. Why do you feel that you can or cannot be of use to society right now?
   *Open-ended question*
8. Indicate whether you agree with the following statement. I would like to do something to help others during this crisis.
   *Possible answers: 1) Entirely agree, 2) Mostly Agree, 3) Somewhat agree, 4) Neither Agree nor Disagree, 5) Somewhat Disagree, 6) Mostly Disagree, 7) Entirely disagree*
9. How would you like to help others during this crisis?
   *Open-ended question*
10. Do you feel that you are at risk to be infected with the coronavirus?
    *Possible answers: 1) Yes, 2) No, 3) I already have (had) the virus, 4) Other: …*
11. Indicate whether you agree with the following statement. I am less willing to help others because I am afraid to be infected with the coronavirus.
    *Possible answers: 1) Entirely agree, 2) Mostly Agree, 3) Somewhat agree, 4) Neither Agree nor Disagree, 5) Somewhat Disagree, 6) Mostly Disagree, 7) Entirely disagree*
12. Did you meet with friends last day in a public place like a parc or mall?
    *Possible answers: 1) Yes, 2) No, 3) Other: …*
13. Did you meet with a friend last day while you were coughing, experiencing a sore throat, a runny nose, or some other health complaint?
    *Possible answers: 1) Yes, 2) No, 3) Other: …*
14. Indicate whether you agree with the following statement. I understand that people are hoarding supermarkets right now.
    *Possible answers: 1) Entirely agree, 2) Mostly Agree, 3) Somewhat agree, 4) Neither Agree nor Disagree, 5) Somewhat Disagree, 6) Mostly Disagree, 7) Entirely disagree*
15. Indicate whether you agree with the following statement. I would at this moment go to a party if my friends were to organize one at home.
    *Possible answers: 1) Entirely agree, 2) Mostly Agree, 3) Somewhat agree, 4) Neither Agree nor Disagree, 5) Somewhat Disagree, 6) Mostly Disagree, 7) Entirely disagree*
16. Indicate whether you agree with the following statement. I think it is important that people stay at home and limit their contact with the outside world.
    *Possible answers: 1) Entirely agree, 2) Mostly Agree, 3) Somewhat agree, 4) Neither Agree nor Disagree, 5) Somewhat Disagree, 6) Mostly Disagree, 7) Entirely disagree*
17. Indicate whether you agree with the following statement. If you are not among the risk groups you do not have to follow the governments’ rules at all.
    *Possible answers: 1) Entirely agree, 2) Mostly Agree, 3) Somewhat agree, 4) Neither Agree nor Disagree, 5) Somewhat Disagree, 6) Mostly Disagree, 7) Entirely disagree*
18. Indicate whether you agree with the following statement. I feel tense and/or uneasy.
    *Possible answers: 1) Entirely agree, 2) Mostly Agree, 3) Somewhat agree, 4) Neither Agree nor Disagree, 5) Somewhat Disagree, 6) Mostly Disagree, 7) Entirely disagree*
19. Indicate whether you agree with the following statement. I am experiencing negative thoughts and ruminate about what is happening in the world.
    *Possible answers: 1) Entirely agree, 2) Mostly Agree, 3) Somewhat agree, 4) Neither Agree nor Disagree, 5) Somewhat Disagree, 6) Mostly Disagree, 7) Entirely disagree*
20. I am among the risk groups that are more prone to become ill because of the coronavirus.
    *Possible answers: 1) Yes, 2) No, 3) Other: …*
21. Are you, your family members, or other people around you infected with the coronavirus?
    *Possible answers (multiple answers possible): 1) Yes, family members/people around me: …, 2) Yes, I am, 3) No*
22. Indicate whether you agree with the following statement. I worry about the family members/people around me who are infected with the coronavirus.
    *Possible answers: 1) Entirely agree, 2) Mostly Agree, 3) Somewhat agree, 4) Neither Agree nor Disagree, 5) Somewhat Disagree, 6) Mostly Disagree, 7) Entirely disagree
    Display logic: Answer to question 17 is 1.*
23. **Profile of Mood States – Vigor Subscale***Questionnaire developed by Wald & Mellenbergh, 1990*

Below you find a list of words. These words describe different moods. We ask you to indicate how much each mood matches the way you currently feel. Don’t think about your answer for too long. This is about your first impression. There are no wrong answers. Every answer can be right, as long as it reflects your own mood. Do not skip any words. This word describes my mood RIGHT NOW: 1) Absolutely not, 2) A little, 3) Neutral, 4) Well, 5) Very well.

1. Active
2. Vigorous
3. Lively
4. Full of pep
5. Cheerful
6. **Profile of Mood States – Tension Subscale***Questionnaire developed by Wald & Mellenbergh, 1990*

Below you find a list of words. These words describe different moods. We ask you to indicate how much each mood matches the way you currently feel. Don’t think about your answer for too long. This is about your first impression. There are no wrong answers. Every answer can be right, as long as it reflects your own mood. Do not skip any words. This word describes my mood RIGHT NOW: 1) Absolutely not, 2) A little, 3) Neutral, 4) Well, 5) Very well.

1. Nervous
2. Panicky
3. Tense
4. Restless
5. Anxious
6. Uneasy
7. **Contributions to Society During the COVID-19 Crisis***Self-developed by van de Groep, Sweijen, Green, Zanolie & Crone (2020)*

Indicate whether you agree with the following statements.

1. I contributed to society last day.
   *Possible answers: 1) Entirely agree, 2) Mostly Agree, 3) Somewhat agree, 4) Neither Agree nor Disagree, 5) Somewhat Disagree, 6) Mostly Disagree, 7) Entirely disagree*
2. I helped others last day.
   *Possible answers: 1) Entirely agree, 2) Mostly Agree, 3) Somewhat agree, 4) Neither Agree nor Disagree, 5) Somewhat Disagree, 6) Mostly Disagree, 7) Entirely disagree*
3. I made an effort for people around me last day.
   *Possible answers: 1) Entirely agree, 2) Mostly Agree, 3) Somewhat agree, 4) Neither Agree nor Disagree, 5) Somewhat Disagree, 6) Mostly Disagree, 7) Entirely disagree*

# **Weekly Measures**

1. **Interpersonal Reactivity Index – Perspective Taking Subscale***Developed by Davis (1983), we used the Dutch version adjusted to be suitable for use with adolescents.*

Select the answer that best matches your opinion. Answer scale: 1) Not true at all, 2) Not true, 3) Neutral, 4) True, 5) Very True

1. When two peers disagree, I try to look at what both of them think.
2. I sometimes try to understand my friends better by imagining how things look from their perspective.
3. I usually understand how peers think about something.
4. I try to look at every side of a disagreement before making a decision.
5. When I’m upset at someone, I usually try to “put myself in their shoes” for a while.
6. Before criticizing somebody, I try to imagine how I would feel if I were in their place.
7. **Interpersonal Reactivity Index – Empathic Concern***Developed by Davis (1983), we used the Dutch version adjusted to be suitable for use with adolescents.*

Select the answer that best matches your opinion. Answer scale: 1) Not true at all, 2) Not true, 3) Neutral, 4) True, 5) Very True

1. I often have tender, concerned feelings for people less fortunate than me
2. Sometimes I don’t feel sorry for other people when they are having problems. **R**
3. When someone is in pain or trouble, I feel anxious and uneasy
4. When peers are having problems, I feel sorry for them
5. When I see someone being treated unfairly, I sometimes don’t feel very much pity **R**
6. I am often quite touched by things that I see happen to peers.
7. **Social Desirability (SDRS-5)***Developed by Hays, Hayashi, & Stewart (1989)*

Indicate whether you agree with the following statements.

1. I am always courteous even to people who are disagreeable
2. There have been occasions when I took advantage of someone **R**
3. I sometimes get even rather than forgive and forget **R**
4. I sometimes feel resentful when I don’t get my way **R**
5. No matter who I’m talking to, I’m always a good listener
6. **Contributions to Society (General)***Self-developed by van de Groep, Sweijen, Green, Zanolie & Crone (2020)*

The following statements are about the extent to which you feel you contribute to society. Contributions to society can take many different forms such as through work, volunteering, or your social contacts.

We would like to ask you to rate on a 10-point scale for every statement (i.e., for each way of contributing) how much you feel you contribute to society. Here, 1 means not at all and 10 means very much.

1. I think it is important to contribute to society a lot.
2. I think my volunteer work is important.
3. I think it is important to make an effort for the people around me.

# **Measures Administered in Start and Final Questionnaire**

1. **Social Value Orientation – Slider***Developed by Murphy, Ackermann, & Handgraaf (2011)*

In this task you will make several decisions about dividing points between yourself and someone else. You can assume that these points are valuable to you both. You and the other person do not know each other and you will stay anonymous for each other. All of your choices are totally confidential. Please click on the distribution of your choice. You can only make one choice per question.


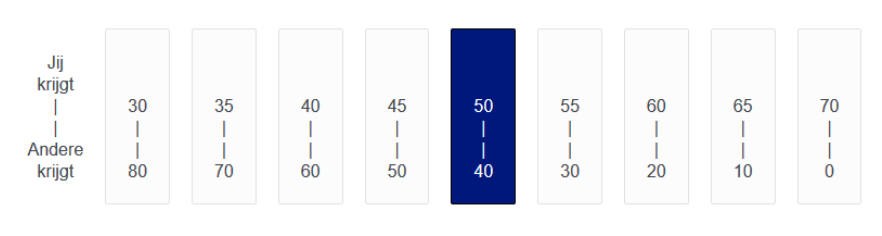
Your decisions will generate points for yourself and the other person. In the example blow, a person choice the distribution in which he/she gets 50 points, while the anonymous other gets 40 points.

You get

Other gets


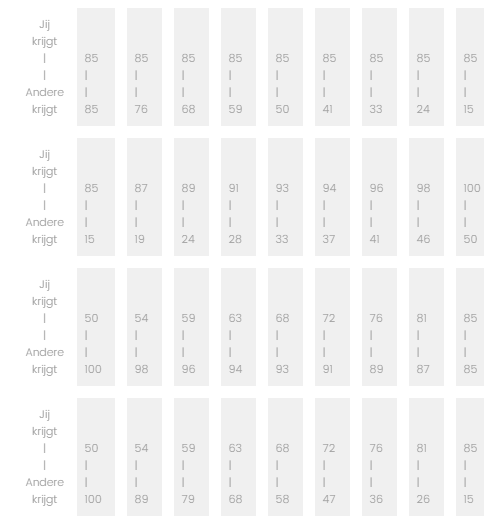


You get

Other gets

You get

Other gets

You get

Other gets

You get

Other gets


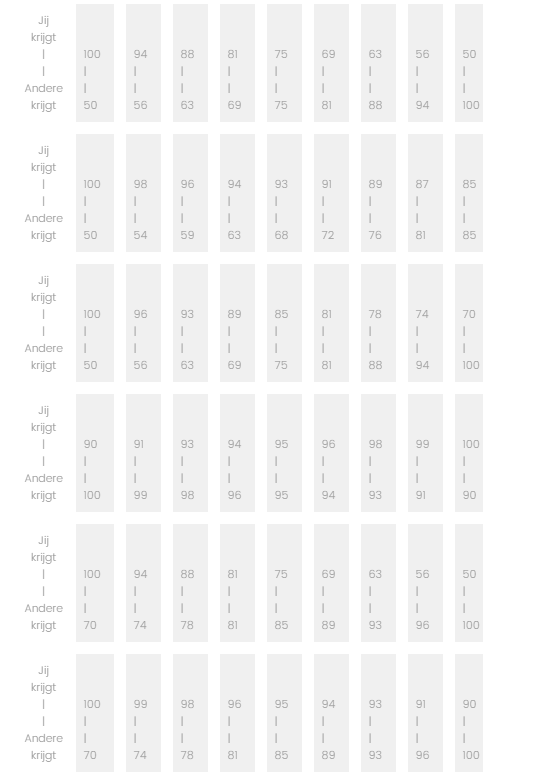


You get

Other gets

You get

Other gets

You get

Other gets


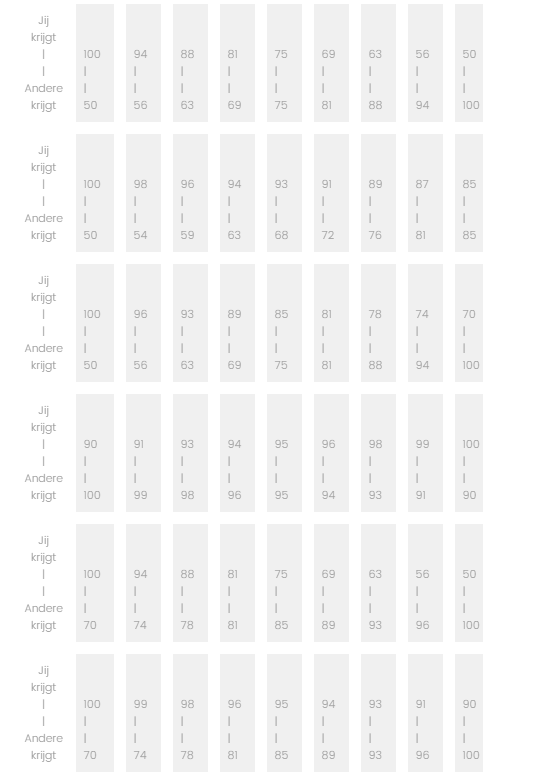


You get

Other gets

You get

Other gets

You get

Other gets


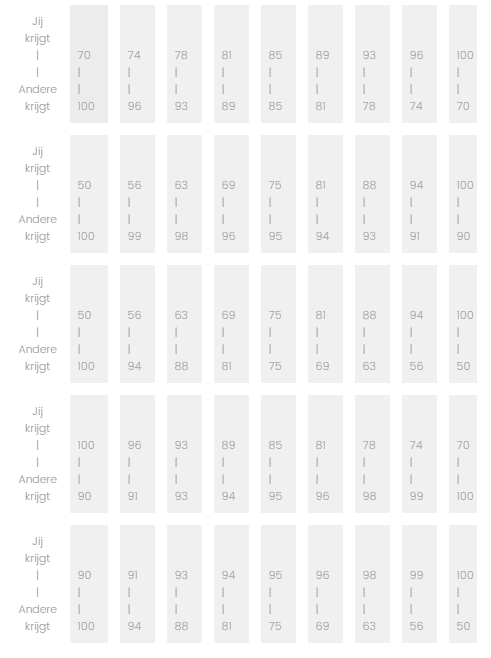


You get

Other gets

You get

Other gets

You get

Other gets

You get

Other gets

You get

Other gets

1. **Dictator Games with Five Targets (in randomised order)***Adapted from Kahneman (1968)*

You will now play a game in which you will divide 10 coins beween yourself and others. You can assume that these coins are valuable to yourself and the other. You can decide yourself how many you want to give to the other and how many you want to keep to yourself. When you keep more coins to yourself, the other has less, and vice versa. There are no right or wrong answers.

You now play with 1) an unknown peer, 2) a friend, 3) a doctor in the hospital, 4) someone who has been infected with the corona virus, 5) someone with a poor immune system. You get 10 coins. How do you divide them between yourself and the other?

1. **Risk-question***Adapted from Risk Propensity Scale (Meertens & Lion, 2008)*

Please indicate how you see yourself on a scale of 0 (risk-avoider) to 100 (risk-seeker). Don’t think about your answer too long, your first hunch is usually best.
