# Supplementary material for: A daily diary study on adolescents’ mood, empathy, and prosocial behavior during the COVID-19 pandemic
Source: PLoS One. 2020 Oct 7;15(10):e0240349. doi: 10.1371/journal.pone.0240349 (PMC7540854; doi:10.1371/journal.pone.0240349)
Supplement: S5 File — (DOCX) [file pone.0240349.s006.docx]

**Overzicht van taken en vragenlijsten voor het project ‘Prosocial Behavior in Adolescence During the Pandemic Covid-19 Crisis’**

*Het huidige document bevat alle taken en vragenlijsten die zijn afgenomen voor ons project ‘Prosocial Behavior in Adolescence During the Pandemic Covid-19 Crisis’. De meetinstrumenten zijn in het Nederlands afgenomen en in dit document terug te vinden. Een vertaling naar het Engels is te vinden op onze OSF pagina. Als je de meetinstrumenten uit dit document wil gebruiken vragen we je om credit te geven aan de originele auteurs. Voor de meetinstrumenten die wij hebben ontwikkeld (e.g. pandemic questionnaire, vragen over bijdragen aan de maatschappij) vragen we dat je ons credit geeft door te refereren naar onze OSF pagina:* [*https://osf.io/kgcdm/*](https://osf.io/kgcdm/)*.*

# **Dagelijkse metingen**

1. **Opportunities for Prosocial Actions – Emotional Support Subschaal***Nog niet gevalideerde vragenlijst, aangepast om aan te sluiten bij de pandemie.*

We willen graag meer weten over de dingen die je voor je vrienden hebt gedaan tijdens de afgelopen dag. Iemand waarmee je een romantische relatie hebt (degene waarmee je verkering hebt) valt hier niet onder.

Straks krijg je vragen over verschillende dingen die je voor anderen kunt doen. Het gaat hier niet om dingen waar je voor betaald werd, of waarvoor je gestraft zou worden als je het niet zou doen. We willen alleen weten wat je uit jezelf hebt gedaan.

Vul hieronder in of je de beschreven dingen voor je vrienden tijdens de afgelopen dag hebt gedaan.

1. Ik heb vandaag vrienden gerustgesteld toen zij overstuur waren.
2. Ik heb vandaag vrienden een berichtje gestuurd, om aardig te zijn.
3. Ik heb vandaag mijn best gedaan om tijd aan vrienden te besteden.

*Ingevuld middels een Likert Schaal: 1) Helemaal niet – 5) Heel veel, en optie: niet van toepassing*

1. **Prosocial Tendencies Measure Revised – Altruism Subschaal***Vragenlijst ontwikkeld door Carlo et al. (2003), aangepast om aan te sluiten bij de pandemie.*

Hieronder staan zinnen die jou wel of niet kunnen beschrijven. Geef alsjeblieft aan in hoeverre elke zin op jou van toepassing is door gebruik te maken van onderstaande schaal. 1) Beschrijft me helemaal niet, 2) Beschrijft me een beetje, 3) Beschrijft me redelijk, 4) Beschrijft me goed, 5) Beschrijft me erg goed.

1. Ik vind één van de beste dingen aan het helpen van anderen dat het mij goed laat lijken. **R**
2. Ik geloof dat het besteden van tijd aan anderen het beste werkt als ik er ook een voordeel uit haal. **R**
3. Ik vind dat ik meer beloningen zou moeten ontvangen voor de tijd en energie die ik besteed aan vrijwilligerswerk. **R**
4. Eén van de beste dingen aan mijn hulp aan anderen is dat het goed overkomt. **R**
5. Ik vind dat als ik iemand help, diegene me zou moeten helpen in de toekomst. **R**
6. Ik help vaak, zelfs als ik denk dat ik er zelf geen voordeel uit kan halen.
7. **Prosocial Tendencies Measure Revised – Dire Subschaal***Vragenlijst ontwikkeld door Carlo et al. (2003)*

Hieronder staan zinnen die jou wel of niet kunnen beschrijven. Geef alsjeblieft aan in hoeverre elke zin op jou van toepassing is door gebruik te maken van onderstaande schaal. 1) Beschrijft me helemaal niet, 2) Beschrijft me een beetje, 3) Beschrijft me redelijk, 4) Beschrijft me goed, 5) Beschrijft me erg goed.

1. Ik ben geneigd mensen te helpen als ze zich in een echte crisis of noodsituatie bevinden.
2. Ik ben geneigd mensen te helpen die erg gekwetst zijn.
3. Het is makkelijk voor me om anderen te helpen als ze zich in een nare situatie bevinden.
4. **Pandemic Questionnaire***Vragenlijst ontwikkeld door van de Groep, Sweijen, Green, Zanolie & Crone (2020)*
5. Zit je momenteel in thuisisolatie? Dat betekent dat je je huis niet verlaat en geen contact hebt met de buitenwereld.
   *Mogelijke antwoorden: 1) Ja, 2) Nee, 3) Anders, namelijk: …*
6. Geef aan of je het eens bent met de volgende stelling: Ik heb het gevoel dat ik op dit moment, tijdens deze crisis, iets kan betekenen voor de maatschappij.
   *Mogelijke antwoorden: 1) Helemaal mee eens, 2) Mee eens, 3) Enigszins mee eens, 4) Niet mee eens, niet mee oneens, 5) Enigszins mee oneens, 6) Niet mee eens, 7) Helemaal niet mee eens*
7. Waarom heb je het gevoel dat je wel of juist niet iets kunt betekenen voor de maatschappij?
   *Open vraag*
8. Geef aan of je het eens bent met de volgende stelling: Ik zou iets willen doen om anderen te helpen tijdens deze crisis.
   *Mogelijke antwoorden: 1) Helemaal mee eens, 2) Mee eens, 3) Enigszins mee eens, 4) Niet mee eens, niet mee oneens, 5) Enigszins mee oneens, 6) Niet mee eens, 7) Helemaal niet mee eens*
9. Hoe zou je anderen willen helpen tijdens deze crisis?
   *Open vraag*
10. Heb je het gevoel dat je risico loopt om het coronavirus op te lopen?
    *Mogelijke antwoorden: 1) Ja, 2) Nee, 3) Ik heb het virus al (gehad), 4) Anders, namelijk: …*
11. Geef aan of je het eens bent met de volgende stelling: Ik ben minder snel bereid om iets voor een ander te doen, omdat ik bang ben om het virus op te lopen.
    *Mogelijke antwoorden: 1) Helemaal mee eens, 2) Mee eens, 3) Enigszins mee eens, 4) Niet mee eens, niet mee oneens, 5) Enigszins mee oneens, 6) Niet mee eens, 7) Helemaal niet mee eens*
12. Heb je de afgelopen dag met vrienden afgesproken op een plek waar meerdere mensen kunnen komen, zoals het park of een winkelcentrum?
    *Mogelijke antwoorden: 1) Ja, 2) Nee, 3) Anders: …*
13. Heb je de afgelopen dag afgesproken met een vriend terwijl je last had van keelpijn, hoesten, een snotneus, of een andere gezondheidsklacht
    *Mogelijke antwoorden: 1) Ja, 2) Nee, 3) Anders: …*
14. Geef aan of je het eens bent met de volgende stelling: Ik begrijp het dat mensen aan het hamsteren zijn in de supermarkt.
    *Mogelijke antwoorden: 1) Helemaal mee eens, 2) Mee eens, 3) Enigszins mee eens, 4) Niet mee eens, niet mee oneens, 5) Enigszins mee oneens, 6) Niet mee eens, 7) Helemaal niet mee eens*
15. Geef aan of je het eens bent met de volgende stelling: Ik zou momenteel naar een huisfeestje gaan als vrienden van mij dat zouden organiseren.
    *Mogelijke antwoorden: 1) Helemaal mee eens, 2) Mee eens, 3) Enigszins mee eens, 4) Niet mee eens, niet mee oneens, 5) Enigszins mee oneens, 6) Niet mee eens, 7) Helemaal niet mee eens*
16. Geef aan of je het eens bent met de volgende stelling: Ik vind het belangrijk dat mensen thuisblijven en nauwelijks fysiek contact hebben met de buitenwereld.
    *Mogelijke antwoorden: 1) Helemaal mee eens, 2) Mee eens, 3) Enigszins mee eens, 4) Niet mee eens, niet mee oneens, 5) Enigszins mee oneens, 6) Niet mee eens, 7) Helemaal niet mee eens*
17. Geef aan of je het eens bent met de volgende stelling: Als je niet in de risicogroep valt hoef je de regels van de overheid niet helemaal te volgen.
    *Mogelijke antwoorden: 1) Helemaal mee eens, 2) Mee eens, 3) Enigszins mee eens, 4) Niet mee eens, niet mee oneens, 5) Enigszins mee oneens, 6) Niet mee eens, 7) Helemaal niet mee eens*
18. Geef aan of je het eens bent met de volgende stelling: Ik heb last van een gespannen en/of onrustig gevoel.
    *Mogelijke antwoorden: 1) Helemaal mee eens, 2) Mee eens, 3) Enigszins mee eens, 4) Niet mee eens, niet mee oneens, 5) Enigszins mee oneens, 6) Niet mee eens, 7) Helemaal niet mee eens*
19. Geef aan of je het eens bent met de volgende stelling: Ik heb last van negatieve gedachtes of piekeren over wat er in de wereld gebeurt.
    *Mogelijke antwoorden: 1) Helemaal mee eens, 2) Mee eens, 3) Enigszins mee eens, 4) Niet mee eens, niet mee oneens, 5) Enigszins mee oneens, 6) Niet mee eens, 7) Helemaal niet mee eens*
20. Ik behoor tot één van de risicogroepen om vatbaarder te zijn voor COVID-19
    *Mogelijke antwoorden: 1) Ja, 2) Nee, 3) Anders: …*
21. Heb jij en/of mensen in je familie/omgeving symptomen van het coronavirus?
    *Mogelijke antwoorden (meerdere antwoorden mogelijk): 1) Ja, mensen in mijn familie/omgeving: …, 2) Ja, ikzelf, 3) Nee*
22. Geef aan of je het eens bent met de volgende stelling: Ik maak me zorgen om de mensen in mijn familie en/of omgeving die symptomen van het coronavirus hebben.
    *Mogelijke antwoorden: 1) Helemaal mee eens, 2) Mee eens, 3) Enigszins mee eens, 4) Niet mee eens, niet mee oneens, 5) Enigszins mee oneens, 6) Niet mee eens, 7) Helemaal niet mee eens
    Display logic: Antwoord op vraag 17 is 1.*
23. **Profile of Mood States – Vigor Subschaal***Vragenlijst ontwikkeld door Wald & Mellenbergh, 1990*

Hierna volgt een lijst met woorden. Deze woorden beschrijven gevoelstoestanden. Het is de bedoeling dat je aangeeft in welke mate de betekenis van het woord past bij je gevoel op dit moment. Denk niet lang na over je antwoord. Het gaat om je eerste indruk. Er bestaan geen foute antwoorden. Elk antwoord is goed, als het je eigen stemming weergeeft. Sla geen woorden over.

De omschrijving past bij mijn gevoel OP DIT MOMENT: 1) Absoluut niet, 2) Een beetje, 3) Middelmatig, 4) Goed, 5) Heel goed.

1. Actief
2. Helder
3. Levendig
4. Vol energie
5. Opgeruimd
6. **Profile of Mood States – Tension Subschaal***Vragenlijst ontwikkeld door Wald & Mellenbergh, 1990*

Hierna volgt een lijst met woorden. Deze woorden beschrijven gevoelstoestanden. Het is de bedoeling dat je aangeeft in welke mate de betekenis van het woord past bij je gevoel op dit moment. Denk niet lang na over je antwoord. Het gaat om je eerste indruk. Er bestaan geen foute antwoorden. Elk antwoord is goed, als het je eigen stemming weergeeft. Sla geen woorden over.

De omschrijving past bij mijn gevoel OP DIT MOMENT: 1) Absoluut niet, 2) Een beetje, 3) Middelmatig, 4) Goed, 5) Heel goed.

1. Zenuwachtig
2. Paniekerig
3. Gespannen
4. Rusteloos
5. Angstig
6. Onzeker
7. **Contributions to Society During the COVID-19 Crisis***Vragenlijst ontwikkeld door van de Groep, Sweijen, Green, Zanolie & Crone (2020)*

Geef aan in hoeverre je het eens bent met de volgende stellingen.

1. Ik heb mij de afgelopen dag ingezet voor de maatschappij.
   *Mogelijke antwoorden: 1) Helemaal mee eens, 2) Mee eens, 3) Enigszins mee eens, 4) Niet mee eens, niet mee oneens, 5) Enigszins mee oneens, 6) Niet mee eens, 7) Helemaal niet mee eens*
2. Ik heb de afgelopen dag anderen geholpen.
   *Mogelijke antwoorden: 1) Helemaal mee eens, 2) Mee eens, 3) Enigszins mee eens, 4) Niet mee eens, niet mee oneens, 5) Enigszins mee oneens, 6) Niet mee eens, 7) Helemaal niet mee eens*
3. Ik heb mij de afgelopen dag ingezet voor de mensen om mij heen.
   *Mogelijke antwoorden: 1) Helemaal mee eens, 2) Mee eens, 3) Enigszins mee eens, 4) Niet mee eens, niet mee oneens, 5) Enigszins mee oneens, 6) Niet mee eens, 7) Helemaal niet mee eens*

# **Wekelijkse meetinstrumenten**

1. **Interpersonal Reactivity Index – Perspective Taking Subschaal***Ontwikkeld door Davis (1983), we hebben een Nederlandse versie gebruikt geschikt voor adolescenten*

Klik het antwoord aan dat het beste bij jouw mening past. Antwoordschaal: 1) Klopt helemaal niet, 2) Klopt niet echt, 3) Ertussen in, 4) Klopt redelijk, 5) Klopt helemaal

1. Ik snap meestal wel hoe leeftijdsgenoten over iets denken.
2. Ik probeer een probleem altijd van verschillende kanten te bekijken.
3. Als ik boos op iemand ben, probeer ik me ook even voor te stellen hoe hij/zij zich voelt.
4. Voordat ik tegen iemand zeg dat hij/zij iets niet goed doet, probeer ik te bedenken hoe het zou voelen als iemand dat tegen mij zou zeggen.
5. Als twee leeftijdsgenoten het niet met elkaar eens zijn probeer ik te kijken wat ze er allebei van vinden.
6. Ik probeer mijn vrienden beter te begrijpen door te bedenken wat zij van iets vinden.
7. **Interpersonal Reactivity Index – Empathic Concern***Ontwikkeld door Davis (1983), we hebben een Nederlandse versie gebruikt geschikt voor adolescenten*
8. Klik het antwoord aan dat het beste bij jouw mening past. Antwoordschaal: 1) Klopt helemaal niet, 2) Klopt niet echt, 3) Ertussen in, 4) Klopt redelijk, 5) Klopt helemaal
9. Ik heb medelijden met leeftijdsgenoten die het minder goed hebben dan ik.
10. Ik heb soms niet zoveel medelijden met leeftijdsgenoten die problemen hebben. **R**
11. Als iemand pijn heeft of in de problemen zit, voel ik me bang en ongemakkelijk.
12. Als leeftijdsgenoten problemen hebben, vind ik dat zielig voor ze.
13. Als iemand oneerlijk wordt behandeld, voel ik soms niet heel veel medelijden. **R**
14. Ik ben iemand die snel medelijden heeft met leeftijdsgenoten.
15. **Social Desirability (SDRS-5)***Ontwikkeld door Hays, Hayashi, & Stewart (1989)*

Kun je aangeven in hoeverre de volgende stellingen waar zijn voor jou?

1. Ik ben altijd aardig en beleefd, zelfs tegen hele vervelende mensen.
2. Er zijn momenten geweest dat ik gebruik heb gemaakt van een ander. **R**
3. Soms probeer ik eerder iemand terug te pakken dan te vergeven en te vergeten. **R**
4. Soms voel ik me een beetje boos als ik mijn zin niet krijg. **R**
5. Het maakt niet uit met wie ik aan het praten ben, ik luister altijd goed.
6. **Contributions to Society (Algemeen)***Ontwikkeld door van de Groep, Sweijen, Green, Zanolie & Crone (2020)*

De volgende stellingen gaan over in hoeverre jij voor jouw gevoel bijdraagt aan de maatschappij. Bijdragen aan de maatschappij kan op verschillende manieren: bijvoorbeeld in je werk, via vrijwilligerswerk of via je sociale contacten.

We willen je vragen om op een schaal van 1 tot 10 bij elke stelling (d.w.z. per manier van bijdragen) aan te geven hoeveel jij voor jouw gevoel bijdraagt aan de maatschappij. Hierbij staat 1 voor helemaal niet en 10 voor heel veel.

1. Ik vind het belangrijk om veel bij te dragen aan de maatschappij
2. Ik vind mijn vrijwilligerswerk belangrijk
3. Ik vind het belangrijk om mij in te zetten voor de mensen om mij heen

# **Meetinstrumenten afgenomen bij de eerste en laatste meting**

1. **Social Value Orientation – Slider***Ontwikkeld door Murphy, Ackermann, & Handgraaf (2011)*

In deze opdracht neem je een reeks beslissingen over verdelingen van punten tussen jou en een andere persoon. Je kunt er vanuit gaan dat deze punten voor jullie allebei waardevol zijn. Jij en deze ander kennen elkaar niet, en jullie blijven anoniem ten opzichte van elkaar. Al jouw keuzes zijn geheel vertrouwelijk. Maak a.j.b. voor elke van de volgende vragen duidelijk welke verdeling je kiest, door de verdeling van jouw keuze aan te klikken. Je kunt slechts één keuze maken per vraag.


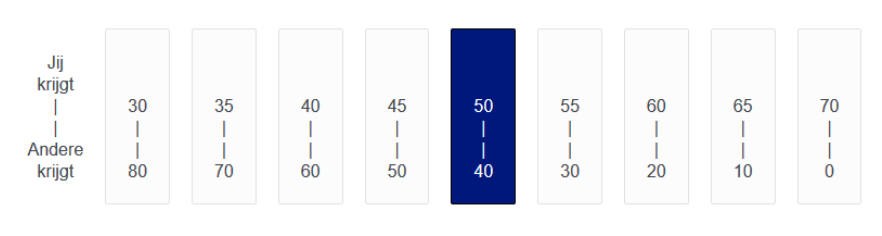
 Jouw beslissingen zullen voor zowel jouzelf als voor de andere persoon punten opbrengen. In het voorbeeld hieronder heeft een persoon gekozen de punten zo te verdelen dat hij/zij 50 punten krijgt, terwijl de anonieme andere persoon 40 punten krijgt.


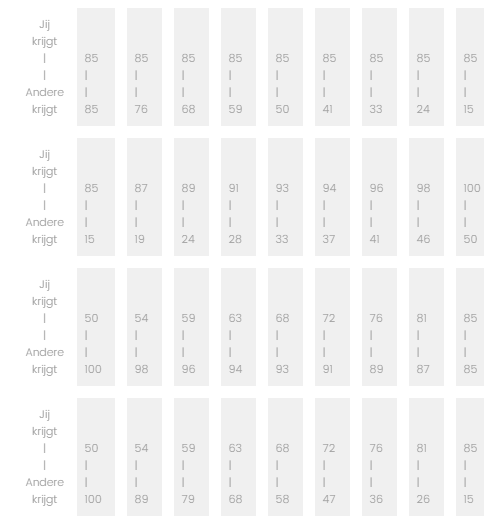


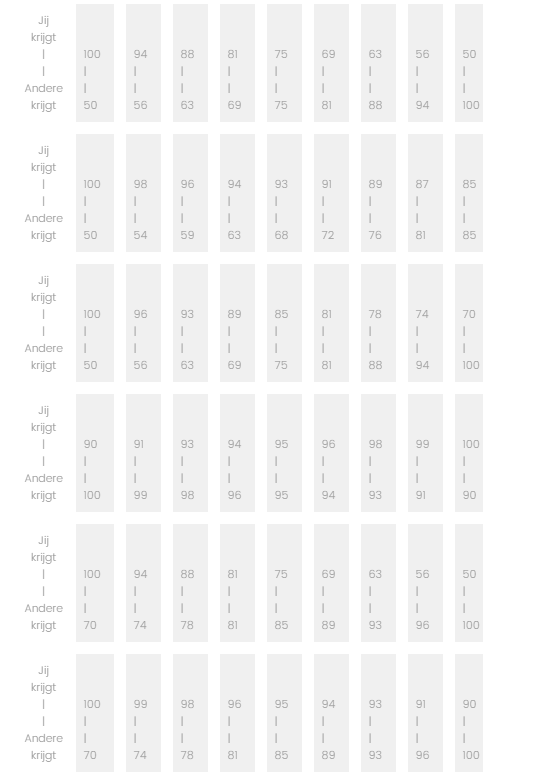


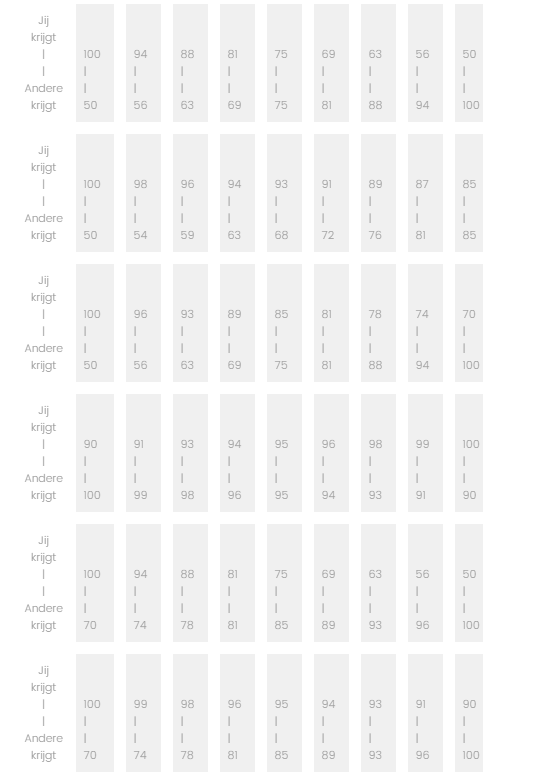


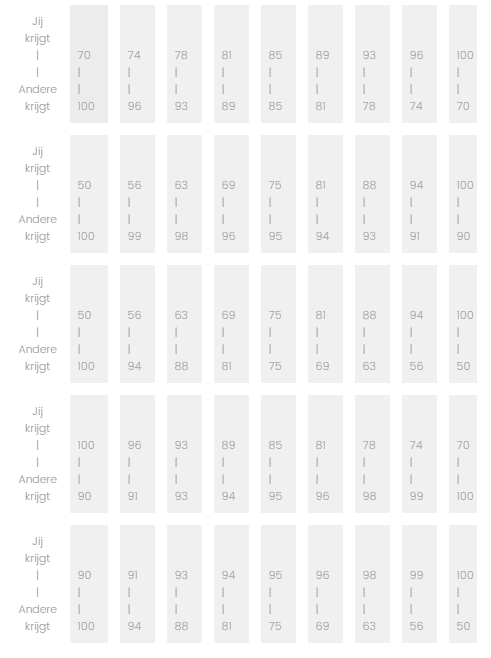


1. **Dictator Games with Five Targets (targets in random volgorde)***Aangepast, gebaseerd op Kahneman (1968)*

Je gaat nu een aantal spellen spelen waarbij je 10 muntjes kunt verdelen tussen jezelf en anderen. Je kunt er vanuit gaan dat deze munten waardevol zijn voor jou en de ander. Je mag zelf kiezen wat je aan de ander wil geven en wat je zelf wil houden. Als je veel muntjes voor jezelf houdt heeft de andere deelnemer minder, en als je meer geeft aan de ander is er uiteindelijk minder voor jezelf. Er zijn geen goede of foute antwoorden.

Je spelt nu met 1) een onbekende leeftijdsgenoot, 2) een vriend, 3) een arts in het ziekenhuis, 4) iemand die het coronavirus heeft, 5) iemand met een lage weerstand. Je krijgt 10 muntjes. Hoe verdeel je ze tussen jezelf en de ander?

1. **Risicovraag***Aangepast op basis van de Risk Propensity Scale (Meertens & Lion, 2008)*

Geef alsjeblieft aan hoe je jezelf ziet op een schaal van 0 (risicovermijder) tot 100 (risicozoeker). Denk niet te lang na over je antwoord, je eerste ingeving is meestal de beste.
