# Supplementary material for: A daily diary study on adolescents’ mood, empathy, and prosocial behavior during the COVID-19 pandemic
Source: PLoS One. 2020 Oct 7;15(10):e0240349. doi: 10.1371/journal.pone.0240349 (PMC7540854; doi:10.1371/journal.pone.0240349)
Supplement: S6 File — (DOCX) [file pone.0240349.s007.docx]

S6. Details on GEE analyses.

# **IRI Perspective taking**

## Number of participants per time point

|  | **N** | **Percent** |
| --- | --- | --- |
| **IRI PT COVID-19** | 53 | 33.8 |
| **IRI_PT_T1** | 51 | 32.5 |
| **IRI_PT_T2** | 53 | 33.8 |
| **Total** | 157 | 100 |

## Goodness of fit

| **Correlation Matrix** | **QIC** |
| --- | --- |
| Independence | 61.914 |
| AR(1) | 61.846 |
| Exchangeable | 61.830 |
| Unstructured | 61.803 |

## Working Correlation Matrix (Unstructured)

|  |  | Measurement | | |
| --- | --- | --- | --- | --- |
|  |  | 1 | 2 | 3 |
| Measurement | 1 | 1.000 | .706 | .733 |
|  | 2 | .706 | 1.000 | .798 |
|  | 3 | .733 | .798 | 1.000 |

##

## Tests of Model Effects

| Wald Chi-Square | df | Sig. |
| --- | --- | --- |
| 12.95 | 2 | .002 |

# **IRI Empathic Concern**

## Number of participants per time point

|  | **N** | **Percent** |
| --- | --- | --- |
| **IRI EC COVID-19** | 53 | 33.8 |
| **IRI_EC_T1** | 51 | 32.5 |
| **IRI_EC_T2** | 53 | 33.8 |
| **Total** | 157 | 100 |

## Goodness of fit

| **Correlation Matrix** | **QIC** |
| --- | --- |
| Independence | 55.193 |
| AR(1) | 55.108 |
| Exchangeable | 55.126 |
| Unstructured | 55.196 |

## Working Correlation Matrix (Autoregressive)

|  |  | Measurement | | |
| --- | --- | --- | --- | --- |
|  |  | 1 | 2 | 3 |
| Measurement | 1 | 1.000 | .743 | .552 |
|  | 2 | .743 | 1.000 | .743 |
|  | 3 | .552 | .743 | 1.000 |

##

## Tests of Model Effects

| Wald Chi-Square | df | Sig. |
| --- | --- | --- |
| 35.29 | 2 | .000 |

# **Opportunities for prosocial actions**

## Number of participants per time point

|  | **N** | **Percent** |
| --- | --- | --- |
| **OPA T1** | 51 | 17.8 |
| **OPA T1.5** | 51 | 17.8 |
| **OPA T2** | 53 | 18.5 |
| **OPA COVID-19 Week 1** | 52 | 18.2 |
| **OPA COVID-19 Week 2** | 39 | 13.6 |
| **OPA Covid-19 Week 3** | 40 | 14.0 |
| **Total** | 286 | 100 |

## Goodness of fit

| **Correlation Matrix** | **QIC** |
| --- | --- |
| Independence | 440.96 |
| AR(1) | 442.42 |
| Exchangeable | 442.68 |
| Unstructured | 445.80 |

## Working Correlation Matrix (Independent)

|  |  | Measurement | | | | | | |
| --- | --- | --- | --- | --- | --- | --- | --- | --- |
|  |  | 1 | 2 | 3 | 4 | 5 | 6 |  |
| Measurement | 1 | 1.000 | .000 | .000 | .000 | .000 | .000 |  |
|  | 2 | .000 | 1.000 | .000 | .000 | .000 | .000 |  |
|  | 3 | .000 | .000 | 1.000 | .000 | .000 | .000 |  |
|  | 4 | .000 | .000 | .000 | 1.000 | .000 | .000 |  |
|  | 5 | .000 | .000 | .000 | .000 | 1.000 | .000 |  |
|  | 6 | .000 | .000 | .000 | .000 | .000 | 1.000 |  |

##

## Tests of Model Effects

| Wald Chi-Square | df | Sig. |
| --- | --- | --- |
| 39.27 | 5 | .000 |

# **Vigor**

## Number of participants per time point

|  | **N** | **Percent** |
| --- | --- | --- |
| **Vigor T1.5** | 43 | 24.7 |
| **Vigor COVID-19 Week 1** | 52 | 29.9 |
| **Vigor COVID-19 Week 2** | 39 | 22.4 |
| **Vigor Covid-19 Week 3** | 40 | 23.0 |
| **Total** | 174 | 100 |

## Goodness of fit

| **Correlation Matrix** | **QIC** |
| --- | --- |
| Independence | 108.75 |
| AR(1) | 108.47 |
| Exchangeable | 108.37 |
| Unstructured | 108.66 |

## Working Correlation Matrix (Exchangeable)

|  |  | Measurement | | | |
| --- | --- | --- | --- | --- | --- |
|  |  | 1 | 2 | 3 | 4 |
| Measurement | 1 | 1.000 | .658 | .658 | .658 |
|  | 2 | .658 | 1.000 | .658 | .658 |
|  | 3 | .658 | .658 | 1.000 | .658 |
|  | 4 | .658 | .658 | .658 | 1.000 |

##

## Tests of Model Effects

| Wald Chi-Square | df | Sig. |
| --- | --- | --- |
| 19.58 | 3 | .000 |

# **Tension**

## Number of participants per time point

|  | **N** | **Percent** |
| --- | --- | --- |
| **Tension T1.5** | 43 | 24.7 |
| **Tension COVID-19 Week 1** | 52 | 29.9 |
| **Tension COVID-19 Week 2** | 39 | 22.4 |
| **Tension Covid-19 Week 3** | 40 | 23.0 |
| **Total** | 174 | 100 |

## Goodness of fit

| **Correlation Matrix** | **QIC** |
| --- | --- |
| Independence | 92.99 |
| AR(1) | 93.53 |
| Exchangeable | 92.97 |
| Unstructured | 92.92 |

## Working Correlation Matrix (Unstructured)

|  |  | Measurement | | | |
| --- | --- | --- | --- | --- | --- |
|  |  | 1 | 2 | 3 | 4 |
| Measurement | 1 | 1.000 | .546 | .652 | .987 |
|  | 2 | .546 | 1.000 | .391 | .606 |
|  | 3 | .652 | .391 | 1.000 | .660 |
|  | 4 | .987 | .606 | .660 | 1.000 |

##

## Tests of Model Effects

| Wald Chi-Square | df | Sig. |
| --- | --- | --- |
| 56.65 | 3 | .000 |

# **Giving at day 1: main effect of target**

## Number of participants per target

|  | **N** | **Percent** |
| --- | --- | --- |
| **Unfamiliar Peer** | 53 | 20 |
| **Friend** | 53 | 20 |
| **Individual with Covid-19** | 53 | 20 |
| **Individual with poor immune system** | 53 | 20 |
| **Doctor in hospital** | 53 | 20 |
| **Total** | 265 | 100 |

## Goodness of fit

| **Correlation Matrix** | **QIC** |
| --- | --- |
| Independence | 655.70 |
| AR(1) | 655.70 |
| Exchangeable | 655.70 |
| Unstructured | 655.70 |

## Working Correlation Matrix (Unstructured)

|  |  | Measurement | | | | |
| --- | --- | --- | --- | --- | --- | --- |
|  |  | 1 | 2 | 3 | 4 | 5 |
| Measurement | 1 | 1.000 | .000 | .000 | .000 | .000 |
|  | 2 | .000 | 1.000 | .000 | .000 | .000 |
|  | 3 | .000 | .000 | 1.000 | .000 | .000 |
|  | 4 | .000 | .000 | .000 | 1.000 | .000 |
|  | 5 | .000 | .000 | .000 | .000 | 1.000 |

##

## Tests of Model Effects

| Wald Chi-Square | df | Sig. |
| --- | --- | --- |
| 169.70 | 4 | .000 |
