# Supplementary material for: A daily diary study on adolescents’ mood, empathy, and prosocial behavior during the COVID-19 pandemic
Source: PLoS One. 2020 Oct 7;15(10):e0240349. doi: 10.1371/journal.pone.0240349 (PMC7540854; doi:10.1371/journal.pone.0240349)
Supplement: S7 File — (DOCX) [file pone.0240349.s008.docx]

**S7. Attrition: did participants who decided not to participate in the daily diary study differ from those who did participate?**

We tested whether participants who decided not to participate in the daily diary study differed from those who did participate in terms of mood and empathic concern prior to the pandemic. ANOVAs with participation in the COVID-19 daily diary study as a predictor (i.e., yes/no) revealed that there were no differences between the groups with regard to perspective taking, age, emotional support, dire prosociality, vigor, and tension on the first measurement (i.e., T1 in 2018 for most variables; T1.5 in 2018-2019 for vigor/tension). There was, however, a significant difference between the groups in empathic concern, F(1, 131) = 4.53, p = .035, such that participants who partook in the daily diary study scored higher (M = 2.68, SD = .59) than those who did not (M = 2.44, SD = .64). The same was observed for altruistic prosociality, F(1, 131) = 4.12, p = .044, such that participants who participated in the daily diary study scored higher (M = 4.28, SD = .59) than those who did not (M = 4.03, SD = .77). Note that these are the exact p-values, uncorrected for multiple comparisons. This suggests that the participants who participated in the daily diary study were slightly more empathic towards friends and slightly more altruistic prior to the pandemic, but did not show differences on the other variables.
