# Supplementary material for: A daily diary study on adolescents’ mood, empathy, and prosocial behavior during the COVID-19 pandemic
Source: PLoS One. 2020 Oct 7;15(10):e0240349. doi: 10.1371/journal.pone.0240349 (PMC7540854; doi:10.1371/journal.pone.0240349)
Supplement: Attachment — Submitted filename: PONE_RevisionSeptember2020_Replyletter_KZ_SvdG.docx [file pone.0240349.s010.docx]

**PONE-D-20-19516R2 Reply Letter - A daily diary study on adolescents’ mood, empathy, and prosocial behavior during the COVID-19 pandemic - PLOS ONE**

**Editor:**
The reviewers still have some minor suggestions before publication. Please address their comments at your earliest convenience. I am looking forward for the final version.

Answer: We thank the editor for the opportunity to revise our article ‘A daily diary study on adolescents’ mood, empathy, and prosocial behavior during the COVID-19 pandemic’. Please find below our replies to the helpful comments we received.

**Reviewers' comments:**

**Reviewer #1:**

**I think the paper improved substantially since the last submission. The authors addressed most of my concerns. I have five suggestion for four changes:

1. “Aim of the study”
In the paper, the authors often write that the “aim of the study was to” i) investigate the effect of the COVID-19 pandemic on adolescents’ mood etc. and ii) investigate the effect of familiarity, need and deservingness on giving behavior. I think this formulation makes most readers belief that these two questions were the initial research questions. However, the study was designed with different research questions in mind (see pre-analysis plan). While the authors mention this in the paper, I think they should avoid saying that “the aim of the study was…” to avoid misunderstandings. (I would prefer, for example, the formulation “this study investigates…”)**

Answer: We completely agree and adjusted our wording throughout the manuscript.

**2. Correction for multiple hypothesis testing
The authors address the concern of multiple hypothesis testing in a separate paragraph. They write that “most confidence should be placed in the results reported with p‐ values < .006 and <
.001, respectively.” The results section does not always follow this recommendation. For example, on p.18 they write ..“comparisons showed that adolescents displayed significantly higher levels of perspective taking during the COVID-19 pandemic (M = 2.74, SD = .63), compared to […] T2 (M = 2.59, SD = .59, p = .040).” Note that this difference is not significant after acounting for multiple hypothesis testing. I think this should be improved. (The authors could, for example, move the discussion of multiple hypothesis testing at the end of the result sections “research aim 1…” and “research aim 2…”, saying that most results are also significant with p‐ values <.006 and <.001, respectively.)**
Answer: We adhered to this suggestion and now mention the correction for multiple hypothesis testing and its implications at the end of the relevant result sections, see p. 19 and p. 20-21.

**3. Small sample size
I am still worried about the small sample size of in between 36 and 53. While the authors can not solve this issue, it might be worth mentioning the sample size in the abstract. This would allow readers that only read the abstract to take the sample size into account when judging the paper.**

Answer: We now mention the sample size (with and without attrition) in the abstract**.**

**4. Section “Peri-pandemic Individual Differences in Giving”
I don’t think the section on “Peri-pandemic Individual Differences in Giving: Influence of …” is very insightful, in particular due to the small sample size. I personally think the paper would improve by deleting this section (or, moving it to the Appendix).**

Answer: We deleted this section and mention in the discussion that future studies should employ larger sample sizes to study individual differences. **5. I think the writing of the paper could be improved**

Answer: We re-read our paper and improved the writing, also based on feedback from a native speaker.

**Reviewer #2:**

**Overview

The study makes multiple, tentatively related observations using a unique survey of adolescents. The authors present one set of findings comparing pre-pandemic and pandemic data: mood improves (vigor increases, tension decreases), empathetic concern decreases, and perspective taking increases. Drawing on data exclusively collected during the pandemic the authors furthermore observe that adolescents state that they would give the same amount of money to healthcare workers as to friends. The authors argue that these results are intimately tied to reduced social contacts during the pandemic.

The authors put great care in the revision, they reacted to my prior comments and rewrote the paper substantially. I do have some remaining and, in my view, relevant concerns detailed below.

1. Selection of individuals into answering different waves of the survey:
a. I would recommend adding the response of the authors on systematic attrition in response to my previous comments in the manuscript (second paragraph of the answer of the authors to the first report, point 6).**

Answer: We included the response to this comment in the manuscript, see page 26-27: “*Second, we invited adolescents enrolled in a cohort-sequential longitudinal study to participate in the daily diary study during the pandemic. While this allowed us to compare experiences and social behavior prior to and during the COVID-19 pandemic, it could have introduced self-selection bias (e.g. inclusion of empathic and altruistic individuals) and it limited our potential sample size. There are several factors that could explain the attrition in this study. First, the pandemic and lockdown resulted in many changes in the lives of adolescents and their parents, including home schooling, new family routines and online communication. The current study was performed ad hoc and required participants to sign up within the period of one week. This may have caused larger attrition than is typical in longitudinal studies. Second, it is noteworthy that attrition was larger for males than for females. There is currently no explanation for this difference, but it is worth noting that other pandemic studies have also shown higher participation of females compared to males [34,35,37,39]. To investigate the extent to which self-selection bias influenced our results, we examined whether adolescents who did not participate in the daily diary study differed from those who did, see S8. These analyses showed that adolescents who participated in the daily diary study showed slightly higher pre-pandemic levels of empathic concern and altruism, but did not differ in terms of mood, perspective-taking, age, and dire prosociality. Future studies should employ large, diverse samples to test the robustness of our findings and to test for individual differences (e.g., in giving)”.*

Details regarding these analyses are provided in supplement 8.

**b. I would recommend showing the main figures capturing the results using two samples: Sample 1 includes all individuals are retained in all surveys (no attrition across points in time, only include people that participated in all surveys). Sample 2 includes every observation independent of whether they were answering all surveys.**

Answer: We thank the reviewer for this suggestion and have changed the main figures to include the sample without attrition (i.e., only including individuals who show no attrition over time) and the sample with attrition (i.e., including all available data per time point).

**2. Standard errors:
a. The authors should state explicitly what assumptions they make to compute the standard errors and corresponding arguments for why these assumptions hold in their data. As far as I understand it, the current analysis assumes that within individual correlations in error terms have the same correlation structure across individuals (that is, errors are independent across individuals, but have a similar/the same correlation within individuals). This may not be the case in the data. In any case, the authors should argue why the assumptions they use to compute standard errors / t-values are reasonable. I also think that the authors should relax the assumptions if they are very restrictive and show the corresponding results (e.g., if the above statement on the error structure applies, what happens if the authors assume that the within-individual dependency of errors are not identical across individuals?).**

Answer: We thank the reviewer for further explaining this point about standard errors. Based on a literature search we now see that RM ANOVAs are relatively inflexible in modeling within-person correlations and that this can lead to biased and less-precise estimates when correlations are unequal across repeated measures (Muth, Bales, Hinde, Maninger, Mendoza, & Ferrer, 2016). This is especially true for small sample sizes, where standard errors are inherently larger. Based on the reviewers’ suggestions, we now performed analyses using generalized estimating equations (GEE). This analysis is suitable for small samples, better equipped to deal with missing data at certain time points, and allows for various types of correlation matrices.

For each variable of interest for which we examined time effects, we inspected which correlation structure provided the best goodness of fit as indicated by the lowest QIC value. Specifically, we tested whether each model was best described by 1) an independent correlation matrix (i.e., zero correlation over time), 2) an exchangeable correlation matrix (i.e., constant correlation over time), 3) an autoregressive correlation matrix (i.e., diminishing correlation over time), and 4) an unstructured correlation matrix (i.e., a freely estimated correlation without constraints. The best fitting model per analysis is mentioned in the manuscript (p. 17 – 20) and further described in S7.

As discussed in Muth et al. (2016): “GEE models are robust to the misspecification of the correlations structure (Zeger & Liang, 1986). Additionally, selecting robust standard errors (Huber/White Sandwich Estimators; as opposed to conventional standard errors) will allow the estimates to be valid even in the event of misspecification of the correlation structure (StataCorp, 2003).” Therefore, we now report robust standard errors throughout the manuscript. Results using GEE models were highly similar to results previously obtained using repeated measures ANOVAs. Nonetheless, as GEE models are more robust and flexible, we decided to report the GEE results in the main text of the manuscript.

**3. Number of emotions:
a. The authors now mention in the text that they only focused on two emotions in the follow-up questionnaires: “Although the original questionnaire included more emotions than vigor and tension, we decided to focus on these subscales as we aimed to focus on a positive emotion (vigor) to get an indication of adolescent resiliency, and a negative emotion (tension) as prior studies have suggested increases in adolescent tension and anxiety as a result of the pandemic (2).” This sentence is not clear to me. It sounds like the authors had more questions in the questionnaire they ended up administering, but I had the impression the authors only included questions on vigor and tension in the questionnaire in the follow-ups. Could the authors clarify which one of these applied? If only vigor and tension were included in the follow-up questionnaires this should be more explicitly stated. If more emotions were collected, this should be added to the statement.**

Answer: Indeed, we measured only vigor and tension in the daily diary study, whereas we measured all emotions of the original POMS questionnaire at T1.5. This is now emphasized on page 14: “*To measure mean levels and fluctuations in vigor and tension, we used the Profiles of Mood States (POMS; [23]). Although the original questionnaire includes more emotions than vigor and tension, these were not measured during the daily diary study (i.e., at T1.5 we did include all emotions as measured by the POMS). We decided to focus only on vigor and tension subscales in the daily diary study to shorten this study’s length and minimize pressure for participants. We included a positive emotion (vigor) to get an indication of adolescent resiliency, and a negative emotion (tension) as prior studies have suggested increases in adolescent tension and anxiety as a result of the pandemic [2].*”
 **Minor points: The last sentence of the abstract could be sharpened. It seems a bit broad at the moment.**

Answer: We changed to last sentences of the abstract to: *“This suggests that during the pandemic need and deservedness had a greater influence on adolescent giving than familiarity. Overall, this study suggests detrimental effects of the first weeks of lockdown on adolescents’ empathic concern and opportunities for prosocial actions, which are important predictors of healthy socio-emotional development and resilience. Conversely, adolescents also showed marked resilience and a willingness to benefit others as a result of the lockdown, as evidenced by improved perspective taking and mood, and high sensitivity to need and deservedness in giving to others.”*
